# Inclusion of Hydroxycinnamic Acids in Methylated Cyclodextrins: Host-Guest Interactions and Effects on Guest Thermal Stability

**DOI:** 10.3390/biom11010045

**Published:** 2020-12-31

**Authors:** Lee E. Hunt, Susan A. Bourne, Mino R. Caira

**Affiliations:** Centre for Supramolecular Chemistry Research, Department of Chemistry, University of Cape Town, Rondebosch 7701, South Africa; trllee001@gmail.com (L.E.H.); susan.bourne@uct.ac.za (S.A.B.)

**Keywords:** cyclodextrins, hydroxycinnamic acids, complexation, thermal analysis, X-ray diffraction, inclusion compounds, self-inclusion, non-inclusion compounds

## Abstract

There is ongoing interest in exploiting the antioxidant activity and other medicinal properties of natural monophenolic/polyphenolic compounds, but their generally low aqueous solubility limits their applications. Numerous studies have been undertaken to solubilize such compounds via supramolecular derivatization with co-crystal formation with biocompatible coformer molecules and cyclodextrin (CD) complexation being two successful approaches. In this study, eight new crystalline products obtained by complexation between methylated cyclodextrins and the bioactive phenolic acids (ferulic, hydroferulic, caffeic, and *p*-coumaric acids) were investigated using thermal analysis (hot stage microscopy, thermogravimetry, differential scanning calorimetry) and X-ray diffraction. All of the complexes crystallized as ternary systems containing the host CD, a phenolic acid guest, and water. On heating each complex, the primary thermal events were dehydration and liberation of the respective phenolic acid component, the mass loss for the latter step enabling determination of the host-guest stoichiometry. Systematic examination of the X-ray crystal structures of the eight complexes enabled their classification according to the extent of inclusion of each guest molecule within the cavity of its respective CD molecule. This revealed three CD inclusion compounds with full guest encapsulation, three with partial guest inclusion, and two that belong to the rare class of ‘non-inclusion’ compounds.

## 1. Introduction

The present study is a sequel to our earlier investigation [1] of the feasibility of using cyclodextrins (CDs, toroidal macrocyclic oligosaccharides with a hydrophobic central cavity and peripheral hydroxyl groups that render these molecules water soluble) to form inclusion complexes with the naturally occurring triphenolic compound *trans*-resveratrol (5-[(1*E*)-2-(4-hydroxyphenyl)ethenyl]-1,3-benzenediol), widely known as a potent antioxidant with inter alia anti-carcinogenic and cardioprotective properties [2,3,4]. Since *trans*-resveratrol (RSV) has very poor aqueous solubility, which severely limits its bioavailability, we were interested in determining whether its complexation with highly soluble methylated CDs might be a viable method for improving its solubility. Furthermore, as there have been no previous reports providing structural information for solid CD complexes of RSV in the literature, it was of interest to attempt to crystallize the target CD·RSV complexes and use X-ray diffraction to establish the nature of host-guest interactions and the detailed mode of inclusion of the RSV molecule in the CD cavity. The study revealed that crystalline inclusion complexes of RSV readily formed with the three CD hosts permethylated α-CD (TMA), permethylated β-CD (TMB), and 2,6-dimethylated β-CD (DMB) via the co-precipitation method. Furthermore, the unique modes of RSV inclusion within the cavities of these host molecules were established for the first time by X-ray diffraction. In the 1:1 inclusion complexes TMA·RSV and DMB·RSV, the 4-hydroxyphenyl ring of RSV was found to enter the respective cavities of the host molecules via their secondary sides, with the phenolic group being connected to the host by a bridging hydrogen-bonded water molecule. The role of water in effecting complexation was noteworthy in these cases. Instead, in the TMB·RSV complex, the phenolic group of RSV was found to be directly hydrogen-bonded to a host primary methoxyl oxygen atom. The three inclusion complexes also displayed unique supramolecular packing features. Finally, phase-solubility studies with DMB and TMB as hosts confirmed the solubility enhancement of RSV and yielded the corresponding complex association constants.

The study described above prompted us to carry out the present analogous investigation with selected bioactive monophenolic and diphenolic compounds, whose chemical structures are shown in Figure S1 (Supplementary Materials). Among these six compounds are the phenolic acids, namely caffeic acid (CAF), ferulic acid (FA), *p*-coumaric acid (PCA), and sinapic acid (SA), which are the most abundant naturally-occurring members of the hydroxycinnamic acid group and are found in significant concentrations in fruits, vegetables, and other plant-based products. A very recent comprehensive review of the hydroxycinnamic acids and their metabolites covered their sources in nature, their pharmacokinetics, biological activities and health benefits [5]. With regard to the biological activities of the hydroxycinnamic acids like the stilbene *trans*-resveratrol referred to earlier, they are primarily potent antioxidants and hence display free radical scavenging ability, and therefore play a preventative role in major medical challenges including cardiovascular disease and cancer. The generally poor aqueous solubility of free hydroxycinnamic acids hinders their development as medicinal agents and they are thus also promising targets for supramolecular derivatization to enhance their delivery properties. Several studies of co-crystallization of hydroxycinnamic acids with biocompatible coformers (e.g., nicotinamide, isonicotinamide) including drug compounds (e.g., caffeine, theophylline, isoniazid, baclofen) have been reported and the crystal structures of numerous co-crystals of this type are documented in the Cambridge Structural Database (CSD) [6]. A review of the significant progress in using the co-crystallization approach to enhance pharmaceutically relevant properties of poorly soluble bioactive compounds has recently appeared [7].

In the present study, however, we attempted to use methylated CDs to create new complexes with the selected hydroxycinnamic acids CAF, FA, PCA, and SA as well as their metabolites HFA (dihydroferulic acid) and HCA (dihydrocaffeic acid) (Figure S1, Supplementary Material). The two metabolites are likewise bioactive, HFA possessing anti-inflammatory activity [8] and HCA antioxidant activity [9]. A CSD search confirmed that no CD complexes containing these potential guest compounds had been reported previously. The aims of the study were the synthesis of the target complexes, analysis of their thermal stability using hot stage microscopy (HSM), thermogravimetric analysis (TGA), and differential scanning calorimetry (DSC), and elucidation of their crystal structures by X-ray diffraction to establish their modes of guest inclusion and the nature of the host-guest interactions. Eight new complexes were isolated and investigated as intended, with the thermal analysis methods indicating the common features of complex dehydration and subsequent release of the guest, followed by decomposition of the host CD. The X-ray analyses led to full elucidation of the complex structures and enabled their subsequent classification based on the extent of guest inclusion in their respective hydrophobic host cavities, the main focus of this report. The isolated complexes displayed a variety of guest inclusion modes, spanning the range from full guest encapsulation through to partial guest inclusion, and finally ‘non-inclusion’ (i.e., the occurrence of self-inclusion of the relevant host CD molecules, leading to their exclusive association and the resultant location of the guest molecules in interstitial sites in the crystals).

## 2. Materials and Methods

### 2.1. Materials

The following hydroxycinnamic acids and their derivatives were purchased from Sigma-Aldrich (Steinheim, Germany) and were used as received: 3,4-dihydroxycinnamic acid (caffeic acid, CAF), 3-methoxy-4-hydroxycinnamic acid (ferulic acid, FA), 4-hydroxycinnamic acid (*p*-coumaric acid, PCA), 3,5-dimethoxy-4-hydroxycinnamic acid (sinapic acid, SA), 3,4-dihydroxyhydrocinnamic acid (hydrocaffeic acid, HCA), and 3-methoxy-4-hydroxyhydrocinnamic acid (hydroferulic acid, HFA). Cyclodextrin (CD) hosts employed in the study included heptakis(2,6-di-*O*-methyl)-β-CD (DIMEB or DMB), heptakis(2,3,6-tri-*O*-methyl)-β-CD (TRIMEB or TMB), and hexakis(2,3,6-tri-*O*-methyl)-α-CD (TRIMEA or TMA).

### 2.2. Complex Preparation

A common procedure was followed for the preparation of the crystalline complexes. In each case, 0.103 mmol of the host compound (DMB, TMB, or TMA) was dissolved in cold (~4 °C) distilled de-ionized water (2–3 mL) and an equimolar amount of the respective bioactive guest compound was added to the solution with vigorous stirring, which continued at the same low temperature for three days. The solution was subsequently filtered, sealed in a vial, and placed in an oven at 50 °C to crystallize.

### 2.3. Thermal Analysis

The thermal profiles of the inclusion complexes were assessed by a combination of hot stage microscopy (HSM), thermogravimetric analysis (TGA), and differential scanning calorimetry (DSC). HSM analyses were performed with a Linkam TH600 hot stage equipped with a TP92 temperature controller (Linkam Scientific Instruments Ltd., Tadworth, Surrey, UK). Crystals were immersed in silicone oil on a cover slip and micrographs were recorded with a real-time Sony Digital Hyper HAD color video camera (Sony, Johannesburg, South Africa) attached to a Nikon SMZ10 microscope (Nikon, Tokyo, Japan). Images were viewed with the Soft Imaging System program analySIS [10]. The TGA and DSC data were analyzed using the TA Instruments software Universal Analysis 2000 for Windows [11].

Samples were placed in alumina crucibles for TGA measurements and a TA-Q500 instrument (TA Instruments, New Castle, DE, USA) was used to record traces at a heating rate of 10 K min^−1^ with a dry N_2_-purge gas flowing at 50 mL min^−1^. DSC traces were recorded on a DSC-Q200 differential scanning calorimeter (TA Instruments, New Castle, DE, USA) with samples in closed Al pans and under the same conditions as for the TGA measurements.

TGA and DSC curves were generally recorded in duplicate. Full structural formulae for the ternary complexes were determined from the water and guest loss masses recorded by TGA.

### 2.4. X-ray Analysis

Powder X-ray diffraction (PXRD) measurements were performed on a Bruker D8 Advance X-ray diffractometer (Bruker AXS GmbH, Karlsruhe, Germany) equipped with a CuKα_1_ X-ray source (λ = 1.5406 Å) and a LYNXEYE 1-D detector. Powder samples were placed on a zero-background sample holder and mounted on a rotating stage. Voltage and current settings were 30 kV and 15 mA, respectively. The 2θ-range scanned was 4°–40° with a step size of 0.01°. Single crystal X-ray diffraction (SCXRD) data were recorded on a Bruker KAPPA APEX II DUO diffractometer (Bruker AXS GmbH, Karlsruhe, Germany) and a Nonius Kappa CCD diffractometer (Nonius BV, Delft, The Netherlands), both using 1.2 kW graphite-monochromated MoKα-radiation (λ = 0.71073 Å). All data were collected at low temperature using Cryostream coolers (Oxford Cryosystems, Oxford, UK). Structures were solved and refined using programs in the SHELX suite [12]. Data reduction and computational software as well as details of structure refinement are provided in the CIF files (see CCDC deposition numbers in the Supplementary Materials). Non-hydrogen atoms were generally treated anisotropically in least-squares refinement, but for disordered atoms, isotropic refinement was adopted. Attempts were made to locate all hydrogen atoms but those associated with water oxygen atoms were frequently disordered and difference Fourier syntheses generally did not reveal their H atoms. For the CDs and their guest molecules, H atoms were placed in idealized positions using a riding model with *U_iso_* values 1.2–1.5 times those of their parent atoms. In some instances, hydrogen bonding requirements enabled placement of H atoms on water oxygens and their inclusion in the models with distance restraints. Non-routine or other unusual features of structure solution and/or refinement are mentioned explicitly in forthcoming sections. The program Lazy Pulverix [13] was used to obtain simulated PXRD patterns of the inclusion complexes based on their refined SCXRD data.

## 3. Results and Discussion

Experiments using the three methylated cyclodextrins (CDs) and the six phenolic acids listed in Section 2.1, intended to produce CD complexes in single crystal form and thus amenable to X-ray structural elucidation, resulted in the formation of eight products. This was considered a sufficiently large series of new supramolecular analogues whose study might yield insights into their thermal behavior and structural features. Table 1 lists the resulting eight host-guest combinations that yielded target complexes and the respective masses of the CDs used with a constant mass (20 mg) of each guest. As described below, while six of the complexes were found to have the same 1:1 host-guest composition as those of the respective starting materials, the remaining two were subsequently shown to have a 2:1 host-guest stoichiometry. All of the complex crystals contained residual water, which was quantified using thermogravimetry.

### 3.1. Thermal Analysis of the Complexes and Their Structural Elucidation by Single Crystal X-ray Diffraction (SCXRD)

All TGA curves for the ternary complexes revealed water loss at relatively low temperatures and guest loss at sufficiently high temperatures, the respective mass losses enabling quantification of both included water and guest content. Full interpretation of the thermal profile of each complex crystal was based on a combination of data obtained using HSM, TGA, and DSC techniques and a comprehensive summary of the thermal data is given in Table 2. For the most part, details of the thermal and X-ray analyses of these eight products are treated systematically below for three distinct groups of complexes that showed certain common structural features ranging from virtually complete guest molecule encapsulation within the host CD cavity to ‘non-inclusion’ complex formation, the latter being characterized by host self-inclusion, with the guest molecules located in interstitial sites of the self-included CD host framework.

#### 3.1.1. ‘Full’ Guest Encapsulation: TMB·HFA, TMB.PCA, and TMA·PCA

**Figure 1:**
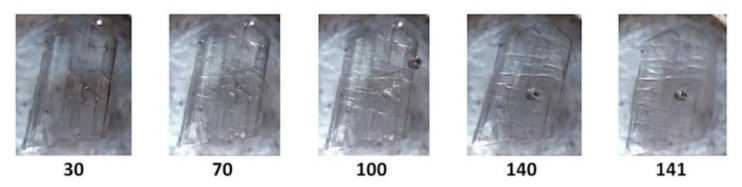
Hot stage microscopy (HSM) micrographs of TMB·HFA (temperatures in °C).

The term ‘full encapsulation’ is used here to signify that in these complexes, the respective guest molecules generally maintain planar conformations and span the entire length of the CD cavity, possibly with only slight protrusion of either or both of their terminal groups (phenolic/carboxylic acid) from the secondary or primary rims of the CD. Results for TMB·HFA and TMB.PCA, which happen to display some degree of isostructurality, are presented first. Initial examination of single crystals of TMB·HFA using HSM (Figure 1) showed the development of fine parallel cracks and the release of small bubbles, indicative of dehydration. Melting commenced at 140 °C.

**Figure 2:**
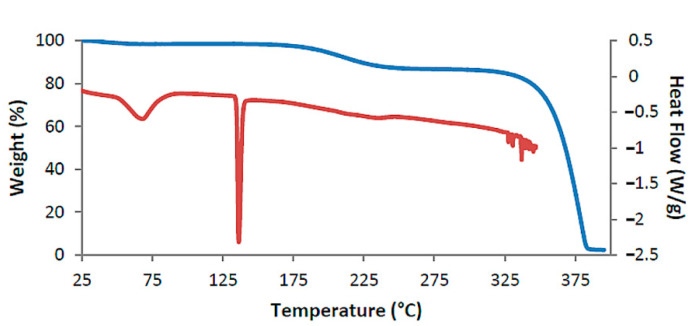
Thermogravimetric analysis (TGA) curve (blue) and DSC curve (red) for TMB·HFA as representative.

Figure 2 shows the TGA and DSC curves for TMB·HFA and the derived data are listed in Table 2. The small water loss (1.4 ± 0.2%) over the 25–100 °C range in the TGA curve corresponded to 1.3 ± 0.2 H_2_O molecules per 1:1 host-guest complex unit. Water loss is evident in the DSC curve as a broad endotherm in the same temperature range. The sharp endotherm peaking at 136 °C corresponded to the melting observed by HSM (Figure 1) and was followed by a broad endotherm at ~230 °C, which signals a one-step mass loss in the TGA curve (T_on_ ~175 °C), representing loss of the guest hydroferulic acid. The measured mass loss of 12.0 ± 0.2% equates to a host-guest (H:G) stoichiometry of 1:(1.0 ± 0.1).

The complex TMB.PCA has a similar thermal profile to that of TMB·HFA except that, with a higher complement of water in the crystal (Table 2), the dehydration process involves two overlapping endotherms followed by a small exotherm (Figure S2, Supplementary Materials), attributed to a phase change of the anhydrous complex. This is in accord with the HSM observation (Figure S3, Supplementary Materials), which indicates crystal fracture and the onset of opacity. This is followed by melting of the new phase at 179 °C and subsequent one-step loss of the guest *p*-coumaric acid. As indicated in Table 2, the complex also has a 1:1 H:G stoichiometry.

**Figure 3:**
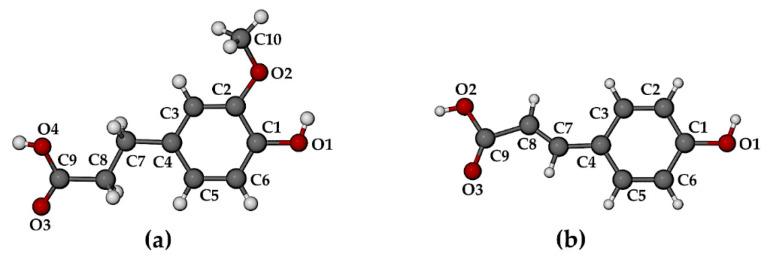
Structures, conformations, and atomic numbering for guest molecules (**a**) hydroferulic acid in the complex TMB·HFA, and (**b**) *p*-coumaric acid in the complex TMB·PCA.

Single crystal X-ray diffraction (SCXRD) indicated that the complexes TMB·HFA and TMB·PCA crystallized in the orthorhombic space group *P*2_1_2_1_2_1_ with similar unit cell dimensions, indicating possible isostructurality of their host frameworks (a common occurrence for CD complexes [14]). With *Z* = 4, their modeled asymmetric units (ASUs) had the respective formulae TMB·HFA(1.3 H_2_O) and TMB·PCA(7.0 H_2_O). Crystal data and details of data-collection and refinement for these complexes are listed in Table S1 (Supplementary Materials). Figure 3 shows the structures, conformations, and atomic numbering for the complexed guest molecules. Non-planar conformations for these molecules are evident, the plane of the carboxylic acid group deviating somewhat from that of the phenyl group, as indicated by the respective torsion angles C7-C8-C9-O4 of −23(1)° for HFA and C3-C4-C7-C8 of 15(2)° for PCA.

**Figure 4:**
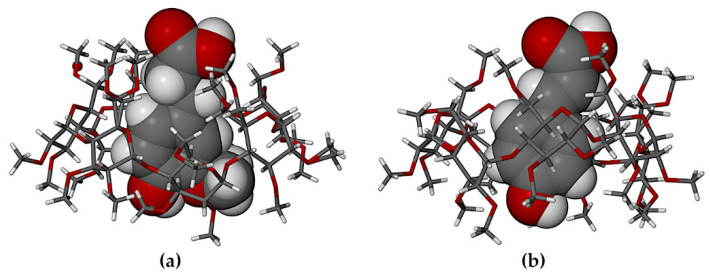
Modes of encapsulation of (**a**) HFA, (**b**) PCA in the host TMB.

In these 1:1 host-guest complexes, the common feature of particular interest is the mode of guest inclusion (Figure 4), namely effectively complete encapsulation of both the HFA and PCA molecules within their respective host cavities, with their carboxylic acid group located near the narrow primary rim of the host (top) and the phenolic groups near the wider secondary rim (bottom).

Geometrical parameters defining the detailed conformations of cyclodextrins are well established [15] and are based on the deviations from regularity of the polygon comprising the glycosidic oxygen atoms (O4) as well as the variations in the angles of tilt of glucose residues relative to the mean plane of the O4-polygon. For the TMB molecule, containing seven glucose rings, the O4-heptagon generally deviates significantly from planarity due to the steric clashes of methyl groups on the secondary rim, which also cause the individual glucose rings to adopt a wide range of tilt angles. For the TMB molecules shown in Figure 4, the definitions of the geometrical parameters and their observed values are listed in Tables S2 and S3 (Supplementary Materials). The view directions in Figure 4 are approximately normal to the respective guest phenyl rings, located in the center of the CD molecule cavities. This common molecular orientation results in an elliptical distortion of each host molecule in the horizontal direction, as a result of the mutual induced fit of host and guest accompanying the inclusion process [16]. The ellipticity of each TMB host molecule is evident from the fact that the values of the parameter *r* (the distance of each O4 atom to the centroid of the O4-heptagon) are not equal, but span relatively wide ranges, namely 4.32–5.50 Å for TMB·HFA and 4.41–5.51 Å for TMB·PCA. The non-planarity of the O4-heptagon is evident from the ranges of values of *α* (the positive or negative distance of each O4 atom from the mean (least-squares) plane through the seven O4 atoms), namely −0.529(3)–0.624(3) Å and −0.668(2)–0.517(3) Å for TMB·HFA and TMB·PCA, respectively. This puckering of the O4-heptagon is accompanied by wide ranges of the tilt angles (*τ_2_*) for the seven permethylated glucose rings, *viz.* 2.5(1)–39.0(1)° (TMB·HFA) and 8.3(1)–36.9(1)° (TMB·PCA).

**Figure 5:**
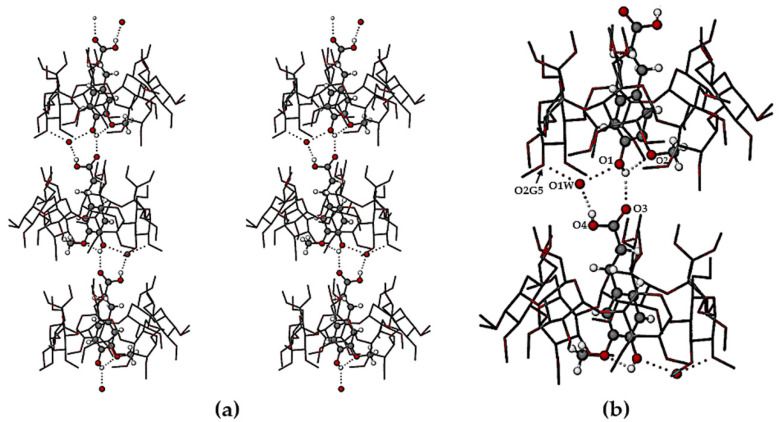
Stereoscopic view of a portion of an infinite column of TMB·HFA complex units with the principal hydrogen bonds (dotted lines) that link the units (**a**) and a magnified view of the hydrogen bonding (dotted lines) (**b**).

The crystal structures of TMB·HFA and TMB·PCA are formed by the close packing of infinite columns of complex units. A representative portion of the column in the TMB·HFA crystal is shown in Figure 5 and an analogous figure for the TMB·PCA crystal is shown in the Supplementary Materials (Figure S4). In these crystals, the complex units comprising the column are related by the twofold screw axis parallel to the crystal *b*-axis. The resulting head-to-tail complex stacking arrangement (Figure 5a) is stabilized by a series of hydrogen bonds, details of which appear in the magnified image (Figure 5b). The principal intermolecular H-bonds in TMB·HFA include a direct guest–guest phenolic(O1-H)···O3(carbonyl) H-bond with O···O 2.753(6) Å and three additional hydrogen bonds mediated by the disordered water molecule O1W, namely carboxylic acid(O4-H)···O1W with O···O 2.692(7) Å, and two inferred H-bonds with the water molecule acting as donor to (a) atom O1 of the phenolic group (O···O 2.696(7) Å) and to (b) a secondary hydroxyl group O atom of the host molecule, O2G5 (O···O 2.793(6) Å). Evident also in Figure 5b is the intramolecular H-bond in the HFA molecule O1-H···O2(methoxyl) with O···O 2.657(9) Å. Analogous head-to-tail guest inclusion of PCA molecules occurred in the crystal of TMB·PCA (Figure S4, Supplementary Materials), but there was no direct H-bonding between successive guest molecules. Instead, the –COOH group of one PCA molecule was linked to the phenolic group of the neighboring PCA molecule via a bridging water molecule. The conformations of the TMB molecules in both TMB·HFA and TMB·PCA were also influenced by numerous intramolecular C–H···O hydrogen bonds. Intermolecular host-host and host-water C–H···O hydrogen bonds in both complexes contribute to their crystal stability. The higher water content in the TMB·PCA complex (7H_2_O per complex unit vs. 1.3H_2_O in the crystal of TMB·HFA) led to a more extensive network of H-bonds.

PXRD patterns for TMB·HFA and TMB·PCA are shown in Figure S5 (Supplementary Material). For each complex, there was good agreement between the experimental pattern and the simulated pattern based on the single crystal X-ray data. In addition, the patterns of the two complexes showed similarities in a few major peak positions due to some degree of isostructurality referred to above.

**Figure 6:**
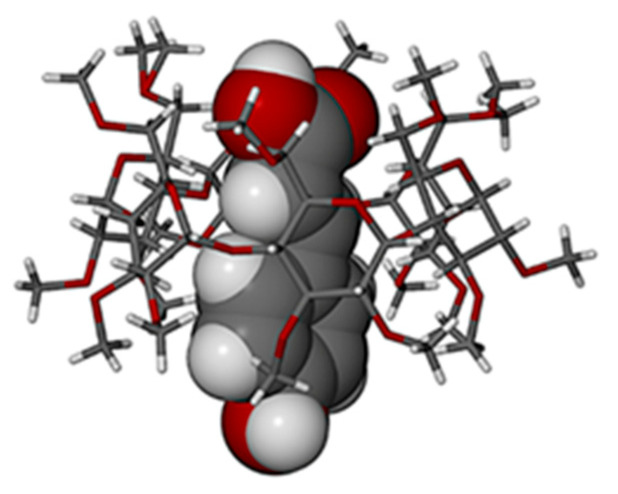
The mode of inclusion of PCA (major disorder component) in the host TMA.

The main features of the thermal analysis results for the crystals of TMA·PCA (the third complex displaying full guest inclusion) were similar to those reported for the previous complexes, namely initial dehydration, melting of the anhydrous phase, and loss of the guest. Salient quantitative thermal data for this complex phase appear in Table 2. The TGA and DSC traces as well as HSM micrographs are shown in Figure S6 (Supplementary Materials) and the crystallographic data for the 1:1 TMA·PCA complex are listed in Table S4 (Supplementary Materials). In this complex, the guest molecule is disordered over two positions (Figure S7, Supplementary Materials), the respective phenyl rings intersecting at an angle of 34.3(5)° and the major component having a site-occupancy factor (s.o.f.) of 0.59(1). The respective disordered –COOH group components were located near the host primary rim, while only the phenolic groups protruded from the secondary side, this inclusion mode (Figure 6) being analogous to those described above for the TMB·HFA and TMB·PCA structures. For clarity, only the major component of the disordered guest is included in Figure 6.

**Figure 7:**
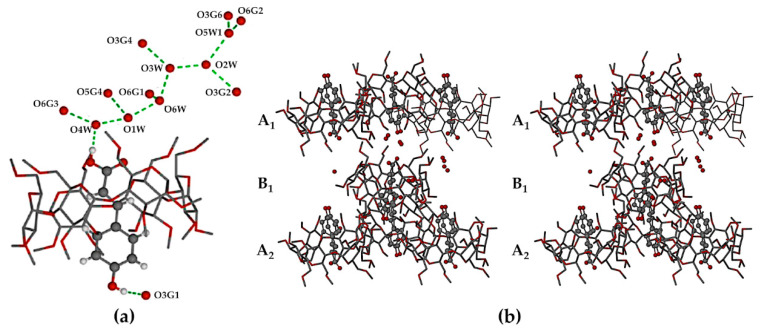
H-bonded network to which the complexed guest PCA molecule (major disorder component) is linked (**a**) and a stereoscopic view of cage-type packing in TMA·PCA (**b**).

The ASU of TMA·PCA contains one complex unit plus 4.5 water molecules, the latter being disordered over eight sites forming a hydrogen bonded network (Figure 7a) that includes methoxyl oxygen atom acceptors (generic labels OmGn) of neighboring host molecules. The network is concentrated in the region above the exposed guest –COOH group, to which it is linked via the H-bond –COOH···O4W. Detailed geometrical parameter data for the host conformation (Table S5, Supplementary Materials) indicate some distortion of the O4-hexagon (parameter *r* range 4.06–4.39 Å, maximum deviation *α* of 0.192(3) Å from the mean O4-plane, *τ_2_* range 5.7(1)–14.4(1)°). In contrast to the previous complexes that associate in infinite columns, the complex units of TMA·PCA form layers parallel to the *ac*-plane with sequence ABAB…, representative complex molecules in three layers are shown in Figure 7b. The representative complex unit in layer B_1_ is sandwiched between three complex units in layer A_1_ and three identical complex units in layer A_2_. This packing mode is similar to that occurring in α-CD cage-type structures [17]. Water molecules are located at the A_1_/B_1_ junction, while the complex units in layers B_1_ and A_2_ are in somewhat closer contact with one another.

The experimental and computed PXRD traces were in good agreement (Figure S8, Supplementary Materials), small differences being due to the different temperatures of intensity data capture for SCXRD and PXRD methods.

#### 3.1.2. Partial Guest Inclusion: DMB·HFA, DMB.PCA, and TMA·FA

In this series of complexes, each guest molecule had one significant residue located within the CD cavity and a significant residue protruding from either the primary or secondary rim of the cavity into the interstitial space. The two DMB complexes showed very similar host packing arrangements and their thermal behaviors are described first.

**Figure 8:**
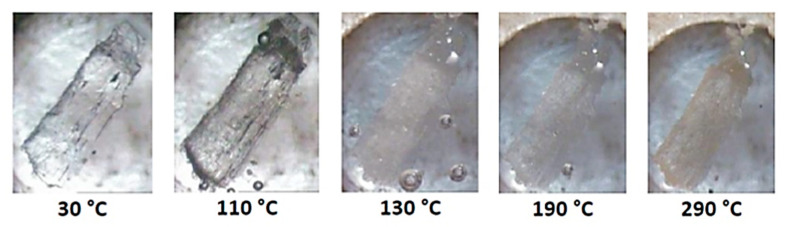
HSM micrographs of DMB·HFA (temperatures in °C).

**Figure 9:**
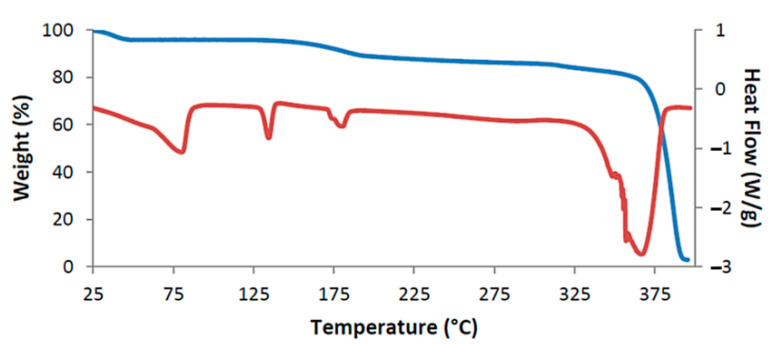
TGA curve (blue) and DSC curve (red) for DMB·HFA as representative.

The successive events on heating a crystal of DMB·HFA immersed in silicone oil are shown in Figure 8. Bubbles initially appear due to dehydration and the crystal becomes opaque on further heating to 130 °C, evidently signifying a phase change (see DSC and TGA behavior in Figure 9). The opacity was reduced at ~190 °C, at which temperature guest loss from the complex crystal commences. A color change observed at ~290 °C indicates decomposition of the host compound.

The assigned dehydration step is seen as a small mass loss in the range 25–100 °C in the TGA curve and as a broad endotherm in DSC (Figure 9). The second endotherm peaking at 134 °C corresponded to the phase change evident in HSM, while the third endotherm, commencing at ~180 °C coincided with the first step of guest release; this was followed by a broad endotherm over the range 225 °C to 325 °C that accompanied the second release.

Figures showing the HSM, TGA, and DSC results for the structurally related complex DMB·PCA appear in the Supplementary Materials (Figure S9). The thermal profiles displayed analogous events to those for DMB·HFA except that the enthalpy values for the corresponding endotherms were significantly smaller. Table 2 lists the quantitative data for DMB·HFA and DMB.PCA derived from their thermal analyses. Both complexes have 1:1 CD:guest stoichiometry and neither displayed melting on HSM.

For the complex TMA·FA (Figure S10, Supplementary Materials; Table 2,) dehydration occurred between 30 and 150 °C. A small endotherm indicates a phase change at 160 °C, which was followed by a sharp endotherm at 185 °C corresponding to fusion of the complex, the latter also being evident in the HSM record. The TGA curve showed complete guest loss in the range 175–300 °C, corresponding to a 1:1 host-guest stoichiometry.

The DMB·HFA and DMB·PCA complexes crystallized in the orthorhombic space group *P*2_1_2_1_2_1_ with similar *a* and *b* unit cell dimensions, but with their *c*-axes differing by ~1.6 Å, suggesting some level of host isostructurality. Their structures were solved by direct methods as attempts to use host atomic co-ordinates from known DMB complexes as trial structures for solution via isomorphous replacement failed. Crystallographic details are listed in Table S6 in the Supplementary Materials.

**Figure 10:**
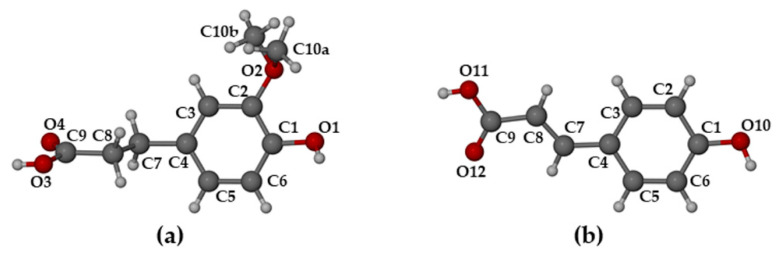
Structures, conformations, and atomic numbering for guest molecules (**a**) hydroferulic acid in DMB·HFA and (**b**) *p*-coumaric acid in DMB·PCA.

The structures of the HFA and PCA molecules in their complexed state with the host DMB are shown in Figure 10.

**Figure 11:**
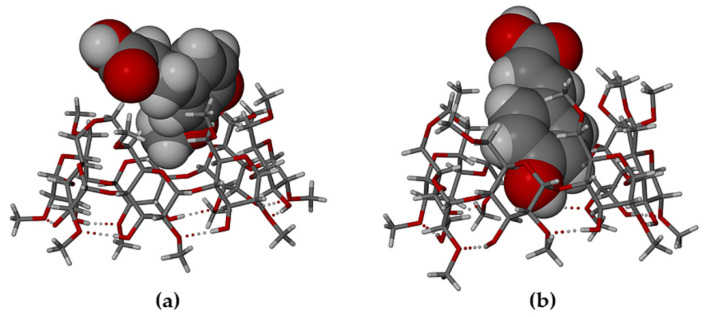
Partial inclusion of (**a**) HFA and (**b**) PCA in the host DMB.

When compared with their counterparts in the complexed state with the TMB molecule (Figure 4 above), it is evident that the HFA molecule adopted a significantly different conformation when it is included in the host DMB (Figure 10a), while the PCA molecule adopted similar conformations when included in both DMB and TMB. For HFA, the torsion angle C3-C4-C7-C8 was −102.5(5)° upon inclusion in DMB and 179.8(8)° when included in TMB. The respective torsion angles in the case of the guest PCA had values of −14.7(1)° and 15(2)°). As shown in Figure 11, both guest molecules were partially included within their respective DMB cavities, but with the larger volume of the HFA molecule protruding from the primary side of the host molecule, rotation of the plane of the carboxylic acid group relative to the phenyl ring was facilitated, enabling the –COOH group to achieve optimum interactions with molecules in the extra-cavity environment. These features are also evident from Figure S11 in the Supplementary Materials, both hosts and guests being drawn in space-filling mode.

**Figure 12:**
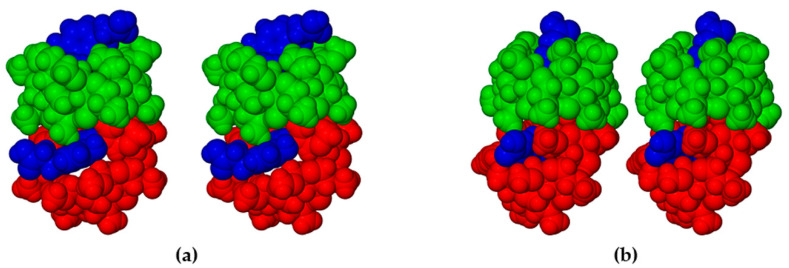
Stereoscopic views showing self-inclusion of representative DMB molecules (green and red for contrast), which limits the cavity space available to the respective guest molecules HFA (**a**) and PCA (blue) (**b**).

Generally, significant differences in the modes of inclusion of a given guest in DMB and TMB may arise from the radically different cavity topologies of these host molecules, the DMB macrocycle typically maintaining a round or slightly elliptical shape due to the belt of seven O2n···H-O3(n-1) hydrogen bonds on its secondary (wider) rim and relatively uniform inclinations of the seven glucose rings (these key features being evident in Figure 11 above), while they are lacking in the TMB molecule (Figure 4). However, Figure 11 above, showing that both the bulk of the HFA molecule and a significant residue of the PCA molecule protrude from their respective host cavities, are deceptive, as the major factor determining their locations within the cavity is instead self-inclusion of the DMB molecules. This is shown in Figure 12, where, for each complex, a portion of one DMB molecule (red) is inserted laterally into the secondary side of the DMB molecule above it (green), thereby limiting the cavity occupation by the guest molecules (blue). Quantitative measures of the extents of self-inclusion are discussed at the end of Section 3.1.3.

The exposed –COOH group and the phenolic group of the HFA guest molecule are hydrogen bonded to distinct interstitial water clusters (Figure S12, left, Supplementary Materials) which are enclosed by neighboring DMB molecules (the latter not shown for clarity). Instead, while the –COOH group of the PCA molecule is likewise hydrogen bonded to an interstitial water cluster, its phenolic group is a donor of a H-bond to oxygen atom O6G2 of the DMB molecule, which partly inserts into the parent host molecule (Figure S12, right, Supplementary Materials). The geometrical parameters for the DMB host molecules in each of these complexes (Tables S7 and S8 in Supplementary Materials) show typical variations indicating their slight ellipticity, the extents of which are guest-dependent. The tilt angle (*τ_2_*) ranges of the glucose residues are somewhat wider for the DMB complex containing the sterically more bulky HFA molecule. Experimental and calculated PXRD traces for the DMB·HFA and DMB·PCA complexes are shown in Figure S13 (Supplementary Materials).

**Figure 13:**
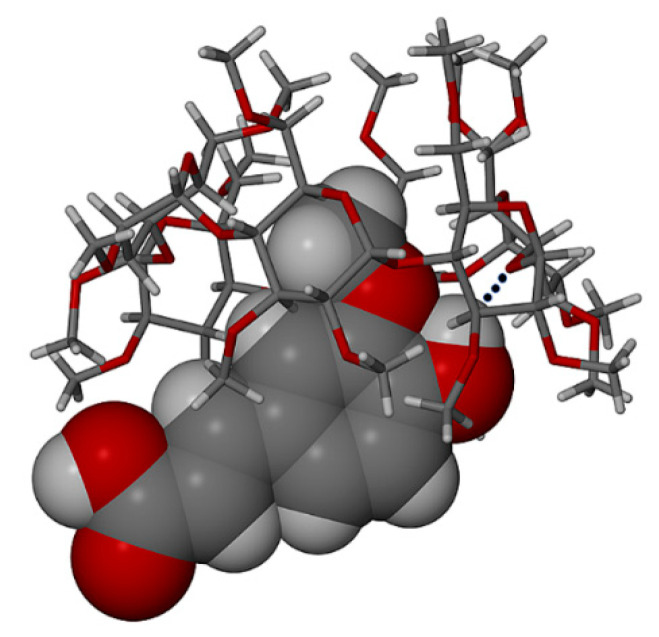
Guest inclusion mode in the complex TMA·FA (H-bond shown as a dotted line).

X-ray analysis of the complex TMA.FA, crystallizing in the monoclinic space group *P*2_1_ with Z = 2 (Table S9, Supplementary Materials), likewise revealed partial guest inclusion. In this case, the FA molecule adopts a planar conformation and is partially included in the host cavity from the secondary side (Figure 13), while the host methoxyl groups on the primary side effectively close the cavity.

The guest molecule is tethered to the host via a hydrogen bond O–H···O (Figure 13) between the included phenolic group and the host glycosidic oxygen atom O4G4 (O····O = 3.031(3) Å) while the carboxylic acid group protrudes from the host secondary rim. This mode of inclusion of FA in TMA contrasts strongly with that of the planar guest molecule PCA in the complex TMA·PCA (Figure 6), which features ‘full’ guest encapsulation. The differences in the conformations of the host TMA molecules in these two complexes, evident from a comparison of the data in Tables S5 (TMA·PCA) and S10 (TMA·FA) (Supplementary Materials) are very significant as a result of both the different modes of guest inclusion as well as a slightly greater steric bulk of the FA molecule, reflecting the flexibility of the common TMA molecule in guest accommodation. In particular, one notes that full encapsulation of PCA is associated with a narrow range of the TMA tilt angles *τ_2_*, namely 3.5–14.4°, while for partial inclusion of FA in TMA, the *τ_2_* range is 7.7–41.4°.

**Figure 14:**
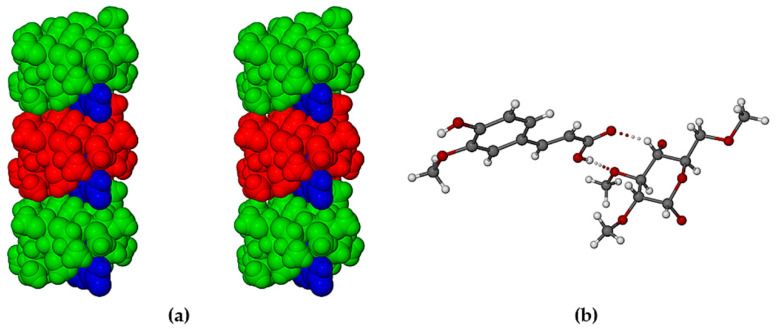
Stereoscopic view of a representative portion of (**a**) a columnar assembly of TMA·FA complexes (TMA molecules in green and red for contrast) with the protruding –COOH groups of the guest FA molecules (blue), and (**b**) O–H···O and C–H···O hydrogen bonds (dotted lines) defining the supramolecular synthon that links each guest –COOH group to a methylglucose residue of a TMA molecule in a neighboring column of complex units.

While partial guest inclusion in the complexes DMB·HFA and DMB·PCA was attributed above to a significant level of host molecule interpenetration (‘self-inclusion’), the latter feature does not apply to the TMA·FA complex. Here, the closure of the primary side of the host TMA molecule by the methoxyl groups and the location of the bulk of the guest molecule within the host cavity leads to stacking of the discrete TMA·FA complex units into infinite columns, a representative portion of a column being shown in the stereodiagram (Figure 14a). The crystal structure is based on anti-parallel columns of complex units that are bound together via the supramolecular heterosynthon shown in Figure 14b. This heterosynthon, with graph-set notation $R_{2}^{2}\left(8\right)$ [18], comprises two hydrogen bonds that link the carboxylic acid group of a guest FA molecule (at *x, y, z*) of one complex column and a O–C–C–H unit of a methylglucose residue (G5) of a TMA molecule (at the symmetry-related position (a) = –x, –1/2 + y, –z) in the neighboring complex column. Details of these interactions are as follows: (1) O4-H···O3G5^a^ with O···O 2.748(3) Å, H···O 1.92 Å, bond angle 170°, and (2) C4G5^a^ -H···O3 with C···O 3.300(4) Å, H···O 2.40 Å, bond angle 149°. These strong intercolumnar links stabilize the complex crystal structure. Additional intercolumnar stabilization is provided by a water molecule (H-O1W-H) that links TMA molecules of neighboring columns via two O–H···O hydrogen bonds. Experimental and calculated PXRD traces for the complex were in good agreement (Figure S14, Supplementary Materials).

#### 3.1.3. ‘Non-Inclusion’ Complexes: DMB·FA and DMB·CAF

**Figure 15:**
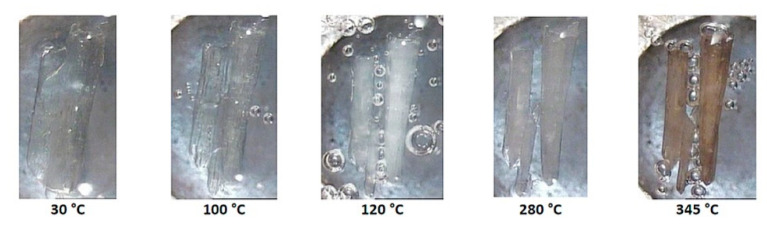
HSM micrographs of DMB·FA (temperatures in °C).

HSM micrographs for the representative complex DMB·FA are shown in Figure 15. Upon heating the initially clear crystal, bubbles accumulate, indicating continued mass loss and the crystal eventually becomes opaque. Discoloration occurs at 280 °C and subsequent vigorous bubble formation indicates final decomposition of the residual material. For the second complex in this category, DMB·CAF, heating results in crystal fracture and at 160 °C, the crystal becomes opaque (Figure S15b, Supplementary Materials). Discoloration from 220 °C indicates decomposition. Again, limited information was available from HSM, but these data complemented the quantitative results from TGA and DSC.

**Figure 16:**
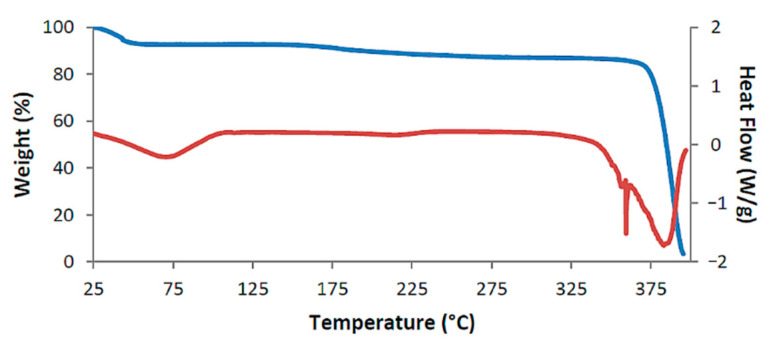
TGA curve (blue) and DSC curve (red) for the representative complex DMB·FA.

Figure 16 shows the combined TGA and DSC traces for the complex DMB·FA while those for DMB·CAF are shown in Figure S15a (Supplementary Materials). Data derived from these traces are listed in Table 2. As with all the previously described thermal profiles, the major events are initial dehydration and guest loss at a significantly higher temperature. For DMB·FA, the initial dehydration mass loss was associated with a broad endotherm spanning the range ~25–120 °C in DSC. A low enthalpy, broad endotherm with onset at 160 °C signals commencement of the guest loss, which is followed by decomposition of the host from ~360 °C. For DMB·CAF (Figure S15b, Supplementary Materials), a small exotherm at 160 °C indicates a possible phase transition of the anhydrous complex, the latter undergoing guest loss from ~210 °C. For both DMB·FA and DMB·CAF, the guest mass losses corresponded to a DMB·guest stoichiometric ratio of 2:1, in contrast to the 1:1 host-guest stoichiometry determined for the previous six complexes. While the thermal analysis results for all eight complexes tended to follow a similar overall profile, with water and guest loss as the primary features, it turned out that the crystal structures of DMB·FA and DMB·CAF differed very significantly from those previously described in this report: as revealed in the next section, they exemplify the extreme situation of ‘non-inclusion’ of the guest molecules in the respective host cavities, a rare feature in the solid-state chemistry of CD complexes.

Single crystal X-ray diffraction indicated very similar unit cell dimensions and a common space group (*P*2_1_) for DMB·FA and DMB·CAF (Table S11, Supplementary Materials), which strongly suggest isostructurality of the host arrangements [14]. Following structural solution of DMB·CAF using phasing from program SHELXD [12], the atomic co-ordinates of the two crystallographically independent host molecules were accordingly used as the trial model for successful structural solution of DMB·FA via isomorphous replacement, the guest molecule and water oxygen atoms being subsequently located in difference Fourier maps.

**Figure 17:**
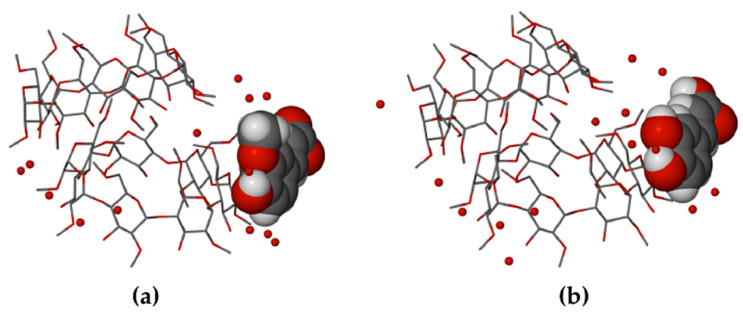
The ASUs in DMB·FA (**a**) and DMB·CAF (**b**). Red spheres represent water oxygen atoms. In (**b**), only the major component of CAF disorder is shown for clarity.

In the crystal of DMB·CAF, the guest molecule is disordered over two positions, the major component having a site-occupancy factor (s.o.f.) of 0.55(1). Minor disorder of the host molecules in these complexes was also modeled appropriately and oxygen atoms of the water molecules were included in the structural models. Each of the asymmetric units (ASUs) in the two complexes (Figure 17) comprised two DMB molecules, one guest molecule and several water molecules (modeled as 11.2 water oxygen atoms occupying 14 sites in DMB·FA, and 11.9 water O atoms occupying 16 sites in DMB·CAF). The predicted isostructural arrangement of the host molecules in the two ASUs was quite evident and some level of isostructurality was also seen to extend to the respective guests and water molecules. As explained in the detailed discussion below, the proper classification of DMB·FA and DMB·CAF as ‘non-inclusion complexes’ emerged as a result of two important structural features evident in Figure 17, namely the significant level of ‘self-inclusion’ displayed by the two DMB molecules, and the location of each guest molecule at the periphery of one of the DMB molecules in the ASU.

**Figure 18:**
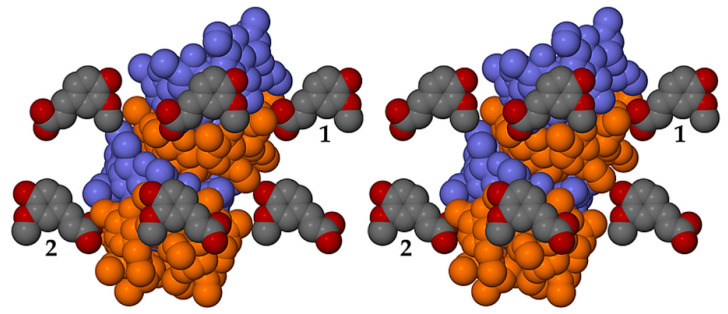
Stereoscopic view of a representative portion of a columnar assembly of DMB host molecules in the DMB·FA complex (alternate DMB molecules in light blue and orange for contrast) showing their self-inclusion. FA guest molecules are located only at the periphery of the columns, those labelled 1 and 2 being screw-axis related, and each being hydrogen bonded via its carboxylic acid group to a DMB secondary oxygen atom acceptor on the column surface (–COOH···O3A4, with O···O 2.637(6) Å). Hydrogen atoms and water oxygen atoms are omitted for clarity.

Application of the space group symmetry (2_1_ parallel to *b*) to each ASU and translation results in infinite spiral columns of self-included DMB molecules parallel to the *b*-axis that preclude guest molecule entry, resulting in guest and water molecules being arranged in a spiral formation at the peripheries of the columns, as shown for the DMB·FA complex as representative (Figure 18).

Complementing the binding of an FA molecule to the surface of the DMB column via its carboxylic acid group, the phenolic group and the methoxyl oxygen atom at the other terminus of the FA molecule engage in H-bonding to chains of water molecules located within the same interstitial channel (Figure S16, Supplementary Materials). These water molecules link the FA molecules to neighboring columns of DMB molecules. Specifically, the phenolic group acts as both H-bond donor to a water oxygen atom [O1-H···O9W, O···O = 2.940(9) Å] and as an H-bond acceptor from a water molecule [O10W-H···O1, O···O = 2.68(1) Å], while the methoxyl atom (O2) is an acceptor of a H-bond from a water molecule [O9W-H···O2, O···O = 2.98(1) Å]. Thus, the FA molecule, being precluded from entry into the host molecule due to the latter’s self-inclusion, is wholly and strongly incorporated within the primarily hydrophilic environment of the interstitial channel, in contrast to the behaviors of the complexes described previously. Analogous observations and remarks apply to the crystal packing displayed by the isostructural complex DMB·CAF.

In view of the rarity of the type of self-inclusion described for these complexes and its possible significance in the context of the well-known phenomenon of CD aggregation, which occurs with increasingly higher CD concentrations in complex solutions [19], further discussion of the geometrical features of this CD association is warranted. As shown in Figure S17 (Supplementary Materials), the two DMB molecules (A and B) in the ASU of the 2:1 DMB·FA complex displayed the phenomenon of ‘self-inclusion’, two methoxyl groups of molecule B entering the cavity of molecule A via the secondary side, with their glucose residues abutting the inner surface of the DMB molecule A. The DMB molecules maintain their usual ‘round’ shape due to the presence of the O2n···H-O3(n-1) hydrogen bonds on their secondary sides [20]. In addition, there is a single hydrogen bond that links the two cyclodextrin molecules directly, namely O3A4-H···O6B6 with H···A 1.85 Å, O···O 2.677(5) Å and bond angle 169°. We considered the following quantitative parameters as being suitable for defining the self-inclusion geometry: (a) the angle between the mean planes of the seven glycosidic (O4) atoms in each of molecules A and B (34.6° in DMB·FA), and (b) the distance between the centroids (Cgs) of the O4 atoms of molecules A and B (two independent distances in DMB·FA, namely CgA··CgB and CgB··CgA′ where A is located at *x, y, z* and A′ at *a, 1 + y, z,* namely 7.86 and 7.82 Å). The corresponding parameters for the closely isostructural complex DMB·CAF are 34.4°, 7.84, and 7.85 Å.

Interesting comparisons can be drawn between the isostructural non-inclusion complexes DMB·FA and DMB·CAF and other ‘irregular’ DMB complexes in the literature. An earlier example of a ‘non-inclusion’ complex is that of the hydrated α-CD complex with 2,5-dihydroxybenzoic acid (DHB), crystallizing in the space group *P*2_1_2_1_2 [21], originally described as a 1:1 α-CD-DHB noncovalent adduct (CSD refcode WIZQEB). In this crystal, the α-CD molecules retain the native CD channel-type packing features that they display in the crystal of the pure host, being aligned in head-to-tail mode, with intermolecular O–H···O hydrogen bonds maintaining the columns (Figure S18, Supplementary Materials). The disordered DHB molecules, however, are located outside the host cavity region and are aligned in hydrogen bonded chains running parallel to the α-CD channel axis, but within the interstitial space created by four neighboring columns of α-CD molecules. Water molecules are also located in the interstitial space and engage in hydrogen bonded networks. This type of ‘non-inclusion’ complex differs very significantly from the type exemplified by the DMB complexes described in the present study, in particular due to the lack of the host ‘self-inclusion’ element.

**Figure 19:**
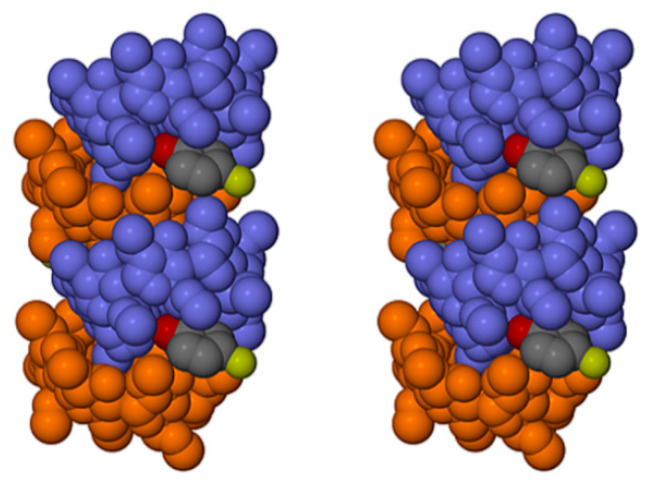
Stereoscopic view of a representative portion of a columnar assembly of DMB host molecules in the DMB·iodophenol complex (alternate DMB molecules in light blue and orange for contrast) showing their self-inclusion. The iodophenol molecule is partially inserted into the extended host cavity formed by the primary methoxyl groups. Hydrogen atoms and water oxygen atoms are omitted for clarity.

The prolific X-ray structural studies of CDs and their complexes published by Harata [22] include those of the isostructural complexes DMB·iodophenol and DMB·*p*-nitrophenol [23], with CSD refcodes DEZMOK10 and DEZMIE10, respectively. Our analysis of the published data show that in these structures, the DMB molecules also associate into columnar assemblies via self-inclusion, but to an extent that still permits simultaneous entry of the guest molecules into successive DMB molecules. This is illustrated in Figure 19 for the iodophenol complex, as representative.

Comparing Figure 18 and Figure 19, the important difference is the absence of guest molecules within the host cavities in the former (showing the DMB·FA complex) due to the ‘tight’ self-inclusion of the DMB molecules that precludes guest entry, while in the latter (showing the DMB·iodophenol complex), the extent of guest inclusion is substantial. Specifically, a significant portion of the iodophenol molecule enters the extended cavity of the DMB molecule, the phenolic group engaging in hydrogen bonding with an intracavity water molecule, while the isostructural DMB·*p*-nitrophenol complex displays analogous features. Such partial guest entry into the DMB cavity reduces the extent of self-inclusion of the DMB molecules, which is reflected in the following quantitative parameters for the DMB·iodophenol structure: (a) the angle between the mean planes of the seven glycosidic (O4) atoms of successive DMB molecules in the column was 48.7° (compared with only 34.6° in DMB·FA), and (b) the distance between the centroids (Cgs) of the O4 atoms of successive DMB molecules in the column was 8.69 Å (compared with only ~7.84 Å in the DMB·FA complex). The above self-inclusion parameters for the DMB·iodophenol crystal were very similar to those calculated for the isostructural DMB·*p*-nitrophenol complex, namely 49.4° and 8.69 Å. Here, we conclude that, despite the common feature of host self-inclusion shown in Figure 18 and Figure 19, the new, isostructural complexes DMB·FA and DMB·CAF reported here are ‘non-inclusion’ complexes while DMB·iodophenol and DMB·*p*-nitrophenol are ‘inclusion’ complexes. The latter complexes in fact resemble the DMB·HFA and DMB·PCA complexes that demonstrate partial guest inclusion (Section 3.1.2) and for which the self-inclusion parameters are respectively 55.9°/8.66 Å, and 58.5°/8.96 Å, the somewhat larger angles that describe the self-inclusion being consistent with deeper guest inclusion within the host cavity.

A variation on the theme is a case of simultaneous inclusion and non-inclusion in a CD-containing crystal, reported by Harata [24], and exemplified by the complex α-CD·(*m*-nitrophenol)_2_ (CSD refcode ACDMNP), crystallizing in the space group *P*2_1_2_1_2. In this crystal, one of the *m*-nitrophenol molecules is included within the host cavity while the other is located at the periphery of the host molecule (Figure S19, Supplementary Materials), occupying a site within an interstitial channel generated by columns of complex units. This complex therefore represents the intermediate case between authentic inclusion complexes (such as DMB·iodophenol, DMB·*p*-nitrophenol) and the authentic non-inclusion complexes DMB·FA and DMB·CAF.

## 4. Conclusions

Of the six bioactive phenolic acids selected as potential guest compounds for inclusion in the methylated cyclodextrins TMA, TMB, and DMB, only FA, CAF, PCA, and HFA yielded complexes via the common procedure employed for their synthesis. These complexes were identified as TMB·HFA, TMB·PCA, TMA·PCA, DMB·HFA, DMB·PCA, and TMA·FA with 1:1 host-guest stoichiometries, and DMB·FA and DMB·CAF with 2:1 host-guest stoichiometries. Thermal analysis enabled determination of both their water contents and guest mass losses and hence their complete formulae, which are listed in the tables of crystal data (Supplementary Materials). However, the thermal stabilities of the complexes reflected in the onset temperatures for guest loss from the crystals spanned the wide range 140–210 °C, from which it was not possible to predict the modes of guest inclusion. Fortuitously, despite the relatively small number of complexes available for analysis, their X-ray structural elucidation revealed a well-defined gradation of guest inclusion modes, the complexes TMB·HFA, TMB·PCA, and TMA·PCA displaying complete guest encapsulation in their respective host cavities and no host self-inclusion, DMB·HFA and DMB·PCA featuring partial guest inclusion in their host cavities with significant host self-inclusion, and the anomalous isostructural pair DMB·FA and DMB·CAF revealing complete self-inclusion of the host molecules that preclude the entry of the guest molecules into the host cavity, resulting in their location in interstitial sites in these crystals. The TMA·FA complex, however, featured no host self-inclusion and partial guest inclusion. The DMB·FA and DMB·CAF complexes were accordingly classified as authentic exemplars of non-inclusion CD complexes. Their unique structural nature was compared with other representative CD complexes that showed some extent of non-inclusion of guest molecules. The existence of the complexes DMB·FA and DMB·CAF thus defies the notion that CD complexation requires guest accommodation within the hydrophobic CD cavity. In the series of eight complexes investigated, only the host DMB demonstrated self-inclusion. Future studies of these complexes will focus on the use of computational methods to gain further insight into the dynamics of CD complexation, and the phenomena of CD self-inclusion and aggregation.

Finally, the term ‘self-inclusion’, used frequently in the present work, refers to the insertion of a residue of one CD molecule into the cavity of a neighboring CD molecule, following Steiner and Saenger, who in 1995 described such a relationship between the CD molecules in a polymorph of DMB [25]. It should be mentioned, however, that another use of the term ‘self-inclusion’ in the CD literature refers to the insertion of a substituent appended to the rim of a derivatized CD molecule into its own cavity, exemplified by another early X-ray diffraction study that revealed full encapsulation of the dansyl moiety within the cavity of the monofunctionalized CD molecule 6-deoxy-6-N-(N’-(5-dimethylamino-1-naphthalenesu1fonyl)diaminoethane)-β-CD [26]. A paper by Barbour et al. [27] provides further perspectives on the term ‘self-inclusion’ in a more general supramolecular chemical context.

## Figures and Tables

**Table 1:** Host-guest pairs forming complexes and the respective component masses.

Guest	CAF	FA	FA	HFA	HFA	PCA	PCA	PCA
Host	DMB	TMA	DMB	TMB	DMB	TMA	TMB	DMB
mg_(guest)_	20	20	20	20	20	20	20	20
mg_(host)_	148	126	137	142	136	149	169	162

**Table 2:** Thermal analysis data and derived results for the complexes.

Complex	% Mass Loss ^1^	*n*H_2_O ^2^	m.p. ^3^ (°C)	T_onset_ (°C) ^4^	% Mass Loss ^4^	H:G Ratio
TMB·HFA	1.4 ± 0.2	1.3 ± 0.2	136	175	12.0 ± 0.2	1: 1.0 ± 0.1
TMB·PCA	6.6 ± 2.2	6.3 ± 2.3	179	210	9.2 ± 0.3	1: 0.9 ± 0.1
TMA·PCA	3.2 ± 1.8	2.5 ± 1.5	200	205	11.6 ± 0.3	1: 1.0 ± 0.1
DMB·HFA	4.2 ± 0.2	3.7 ± 0.2	-	140	13.2 ± 0.3	1: 1.1 ± 0.1
DMB·PCA	3.9 ± 0.7	3.4 ± 0.6	-	210	10.5 ± 0.2	1: 1.0 ± 0.1
TMA·FA	1.3 ± 0.2	1.3 ± 0.1	185	190	13.8 ± 0.2	1: 1.0 ± 0.1
DMB·FA	6.5 ± 1.1	11.0 ± 2.1	-	160	6.5 ± 0.2	2:.1.0 ± 0.1
DMB·CAF	8.9 ± 3.4	15.4 ± 6.4	-	208	6.2 ± 0.4	2: 1.0 ± 0.1

^1^ Complex dehydration; ^2^ Number of water molecules per host-guest complex unit (1:1 or 2:1); ^3^ Fusion of the anhydrous complex; ^4^ For guest loss event.

## Data Availability

Not applicable.

## Supplementary Materials

The following are available online at https://www.mdpi.com/2218-273X/11/1/45/s1, Figure S1: The chemical structures of the phenolic acids selected for study of their propensity as guests for inclusion in methylated cyclodextrins, Figure S2: TGA curve (blue) and DSC curve (red) for TMB·PCA, Figure S3: HSM micrographs of TMB·PCA (temperatures in °C), Figure S4: Stereoscopic view of a portion of an infinite column of TMB·PCA complex units with the principal hydrogen bonds (dotted lines) that link the units (a) and a magnified view of the hydrogen bonding (dotted lines) (b), Figure S5: Experimental and calculated PXRD patterns for TMB·HFA and TMB·PCA, Figure S6: TGA and DSC traces (a) and HSM micrographs (b) for the complex TMA·PCA, Figure S7: Disorder of the PCA molecule in the TMA·PCA complex, Figure S8: Experimental and calculated PXRD patterns for the complex TMA·PCA, Figure S9: TGA and DSC traces (a) and HSM micrographs (b) for the complex DMB·PCA, Figure S10: TGA and DSC traces (a) and HSM micrographs (b) for the complex TMA·FA, Figure S11: CPK space-filling representation of the inclusion complexes DMB·HFA (left) and DMB·PCA (right) viewed from the (narrow) primary sides of the host molecules, Figure S12: Principal H-bonds in DMB·HFA (left) and DMB·PCA (right). Red circles labelled OnW represent the oxygen atoms of water molecules. Atoms with labels of type OpGq (p = serial number, q in Gq = Glucose residue number q) are oxygen atoms of the host molecules. [Atom O6G2 (top right) is an acceptor of the hydrogen atom of the phenolic group of PCA. O6G2 belongs to a DMB molecule (not shown) that is partly included in the parent DMB molecule shown], Figure S13: Experimental and calculated PXRD patterns for DMB·HFA and DMB·PCA, Figure S14: Experimental and calculated PXRD patterns for TMA·FA, Figure S15: TGA and DSC traces (a) and HSM micrographs (b) for the complex DMB·CAF, Figure S16: Packing in DMB·FA showing the interstitial channel created by four spiral columns in which the guest molecule FA and water molecules are located. The contents of the arbitrary square drawn on the left are magnified on the right. The view direction deviates slightly from being parallel with the crystal *b*-axis to reduce atomic overlap. Green spheres represent water oxygen atoms that are not directly H-bonded to the CDs, Figure S17: Stereodiagram showing the inclusion of two primary methoxyl groups of DMB molecule B into the cavity of molecule A via the secondary rim. For clarity, the only H atoms shown are those involved in H-bonding, namely those of type O2n···H-O3(n-1), maintaining the round shape of the DMB molecules, and the strong H-bond O3A4-H···O6B6, linking the two CD molecules directly, Figure S18: The [100] projection of the crystal structure of the complex α-CD·2,5-dihydroxybenzoic acid (refcode WIZQEB). The guest molecules are located in the interstitial space created by the surrounding columns of α-CD cmolecules. The isolated red spheres are oxygen atoms of water molecules, Figure S19: Simultaneous guest inclusion and non-inclusion in the α-CD·(*m*-nitrophenol)_2_ complex (CSD refcode ACDMNP). CCDC numbers: 2046950, 2046952, 2046953, 2046954, 2046955, 2046956, 2046957, 2046958 contain the supplementary crystallographic data for the crystal structure refinements. Table S1: Crystallographic data for TMB·HFA and TMB·PCA, Table S2: Geometrical parameters of the TMB molecule in the complex TMB·PCA, Table S3: Geometrical parameters of the TMB molecule in the complex TMB·HFA, Table S4: Crystallographic data for TMA·PCA, Table S5: Geometrical parameters of the TMA molecule in the complex TMA·PCA, Table S6: Crystallographic data for DMB·HFA and DMB·PCA, Table S7: Geometrical parameters of the DMB molecule in the complex DMB·HFA, Table S8: Geometrical parameters of the DMB molecule in the complex DMB·PCA, Table S9: Crystallographic data for TMA·FA, Table S10: Geometrical parameters of the TMA molecule in the complex TMA·FA, Table S11: Crystallographic data for DMB·CAF and DMB·FA. These data can be obtained free of charge via http://www.ccdc.cam.ac.uk/conts/retrieving.html (or from the CCDC, 12 Union Road, Cambridge CB2 1EZ, UK; Fax: +44-1223-336-033; Email: deposit@ccdc.cam.ac.uk).
