# Supplementary material for: Inclusion of Hydroxycinnamic Acids in Methylated Cyclodextrins: Host-Guest Interactions and Effects on Guest Thermal Stability
Source: Biomolecules. 2020 Dec 31;11(1):45. doi: 10.3390/biom11010045 (PMC7823409; doi:10.3390/biom11010045)
Supplement: Supplementary file 1 [file biomolecules-11-00045-s001.pdf]

SUPPLEMENTARY MATERIAL

for the manuscript

Inclusion of hydroxycinnamic acids in methylated cyclodextrins: host-guest interactions and effects on guest thermal stability

Lee E. Hunt, Susan A. Bourne and Mino R. Caira

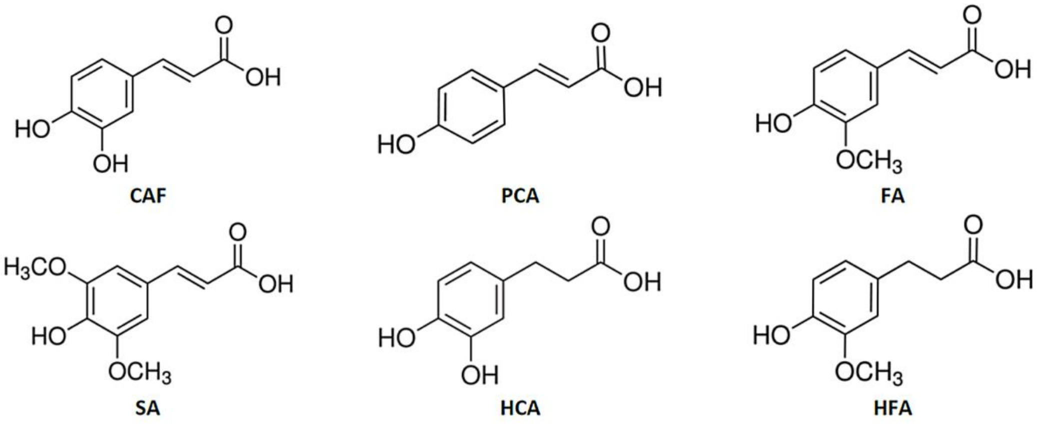

Figure S1. The chemical structures of the phenolic acids selected for study of their propensity as guests for inclusion in methylated cyclodextrins.

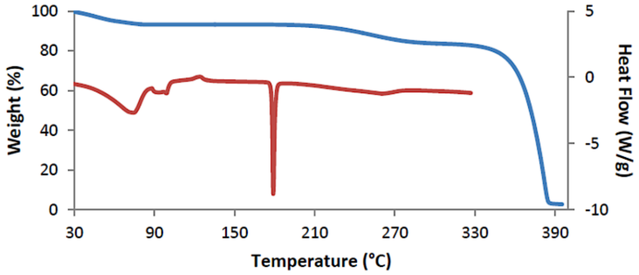

Figure S2. TGA curve (blue) and DSC curve (red) for TMB·PCA.

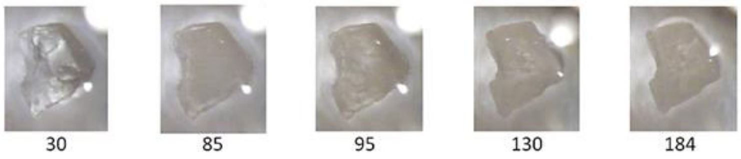

Figure S3. HSM micrographs of TMB·PCA (temperatures in °C).

Table S1. Crystallographic data for TMB HFA and TMB PCA

	**TMB·HFA**	**TMB·PCA**
**Complex Formula**	$C_{63}H_{112}O_{35}$·$C_{10}H_{12}O_4$·1.3H_2O	$C_{63}H_{112}O_{35}$·$C_9H_8O_3$·7H_2O
Formula weight	1649.14	1719.78
Crystal system	Orthorhombic	Orthorhombic
Space group	$P2_12_12_1$ (no. 19)	$P2_12_12_1$ (no. 19)
a / Å	14.657(2)	14.778(3)
b / Å	22.774(3)	22.231(4)
c / Å	26.124(4)	27.728(6)
Volume / $Å^3$	8720(2)	9110(3)
Z	4	4
Calculated density / g cm^{-3}	1.254	1.244
μ (MoKα) / mm^{-1}	0.102	0.104
F (000)	3548	3648
Temperature / K	173(2)	173(2)
Crystal size / mm^3	0.25 x 0.36 x 0.48	0.18 x 0.20 x 0.40
Theta range scanned / °	1.6 < θ < 27.2	1.8 < θ < 26.4
Index ranges	h: -18: 18; k: -29: 28; l: -33: 32	h: -11: 20; k: -19: 24; l: -27: 32
Total number of reflections	57888	97292
No. of independent reflections	19215	18599
No. of reflections with I > 2σ(I)	14183	15850
No. of parameters	1068	969
R_{int}	0.045	0.032
R_1(I > 2σ(I))	0.0697	0.0663
wR_2(I > 2σ(I))	0.2011	0.1912
S	1.028	1.035
Coefficients in weighting scheme	a = 0.0992, b = 6.7909	a = 0.1113, b = 6.7606
Δρ excursions / e $Å^{-3}$	-0.62, 0.78	-0.52, 0.82

Table S2. Geometrical parameters of the TMB molecule in the complex TMB·PCA.**

Residue	r (Å)	D (Å)	a (°)	φ (°)	d (°)	D_3 (Å)	α (Å)	τ_1 (°)	τ_2 (°)
G1	4.551	4.607	140.8	115.8(3)	-9.0	3.374	0.032(2)	24.9(1)	36.9(1)
G2	4.823	4.271	128.2	117.4(3)	14.6	3.212	0.517(3)	19.1(1)	10.1(1)
G3	5.339	4.409	122.6	118.9(4)	11.8	3.241	-0.158(3)	7.6(1)	8.3(1)
G4	5.204	4.353	119.1	118.9(3)	-34.0	3.489	-0.573(2)	19.8(1)	32.8(1)
G5	4.412	4.498	140.5	114.5(3)	7.4	3.833	0.473(2)	43.9(2)	36.6(1)
G6	5.069	4.311	125.2	117.6(3)	22.1	3.725	0.377(2)	15.8(1)	15.1(1)
G7	5.506	4.256	112.1	120.1(3)	-21.5	3.450	-0.668(2)	20.7(1)	32.8(1)
**Mean**	**4.99**	**4.39**	**126.9**	**117.6**	**19.3**	**3.47**	**0.453**	**21.7**	**24.7**

Table S3. Geometrical parameters of the TMB molecule in the complex TMB·HFA.**

Residue	r (Å)	D (Å)	a (°)	φ (°)	d (°)	D_3 (Å)	α (Å)	τ_1 (°)	τ_2 (°)
G1	4.322	4.560	141.9	115.0(5)	16.1	3.731	-0.529(3)	44.9(3)	25.0(2)
G2	4.806	4.185	129.7	118.3(4)	21.5	3.889	-0.204(3)	13.0(1)	25.6(2)
G3	5.496	4.195	113.9	118.6(3)	-23.2	3.355	0.624(3)	19.1(1)	39.0(1)
G4	4.871	4.597	131.7	115.9(3)	-3.0	3.432	-0.160(3)	13.5(1)	27.6(1)
G5	4.585	4.494	133.9	116.5(3)	15.1	3.261	-0.409(3)	17.8(1)	2.5(1)
G6	5.239	4.250	124.1	118.1(4)	7.4	3.096	0.245(3)	6.0(1)	16.1(1)
G7	5.416	4.218	114.5	119.2(4)	-33.7	3.366	0.434(3)	16.3(1)	27.9(1)
**Mean**	**4.96**	**4.36**	**127.1**	**117.4**	**19.6**	**3.45**	**0.406**	**18.7**	**23.4**

** The geometrical parameters tabulated above are defined as follows:

r, the distance of each O4 atom from the centroid of the O4-polygon;
D, the glycosidic O4(n)···O4(n+1) distance;
a, the O4(n-1)···O4(n)···O4(n+1) angle;
ϕ, the intersaccharidic angle C1(n-1)-O4(n)-C4(n);
d, the O4(n)···O4(n+1)···O4(n+2)···O4(n+3) torsion angle;
D_3, the O3(n)···O2(n+1) intra-ring distance;
α, the deviation of each O4 atom from the mean O4-plane;
τ_1, tilt angle: the angle between the plane containing the atoms C1, C2, C3, C4, O5 and C6 of a given glucose ring and the mean O4-plane;
τ_2, tilt angle: the angle between the plane containing the atoms O4(n), C4(n), C1(n) and O4(n+1) of a given glucose ring and the mean O4-plane.

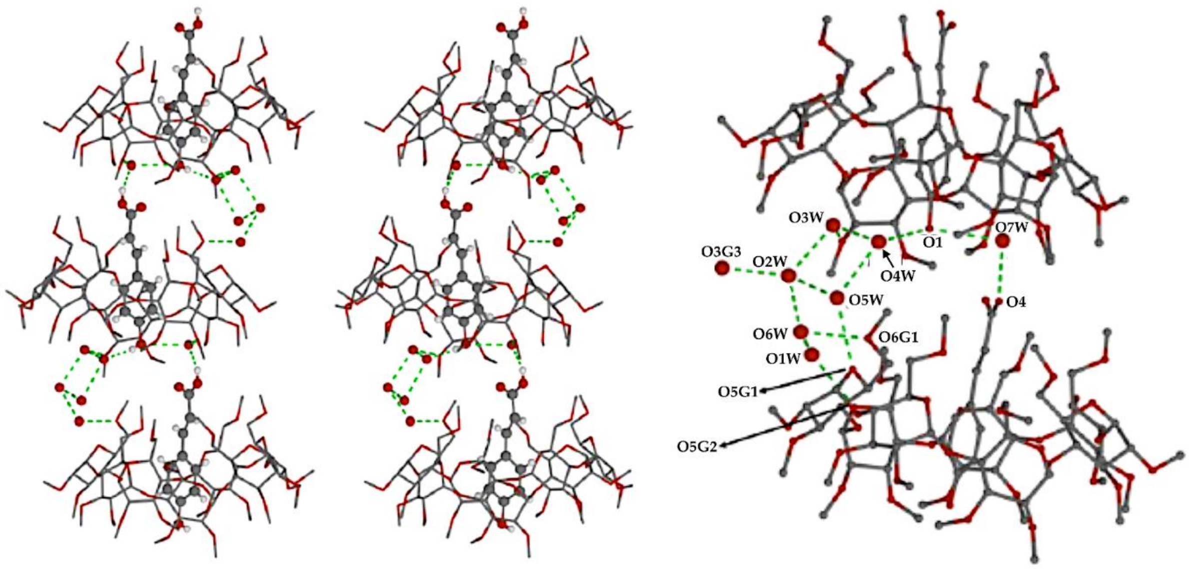

(a) (b)

Figure S4. Stereoscopic view of a portion of an infinite column of TMB ·PCA complex units with the principal hydrogen bonds (dotted lines) that link the units (a) and a magnified view of the hydrogen bonding (dotted lines) (b).

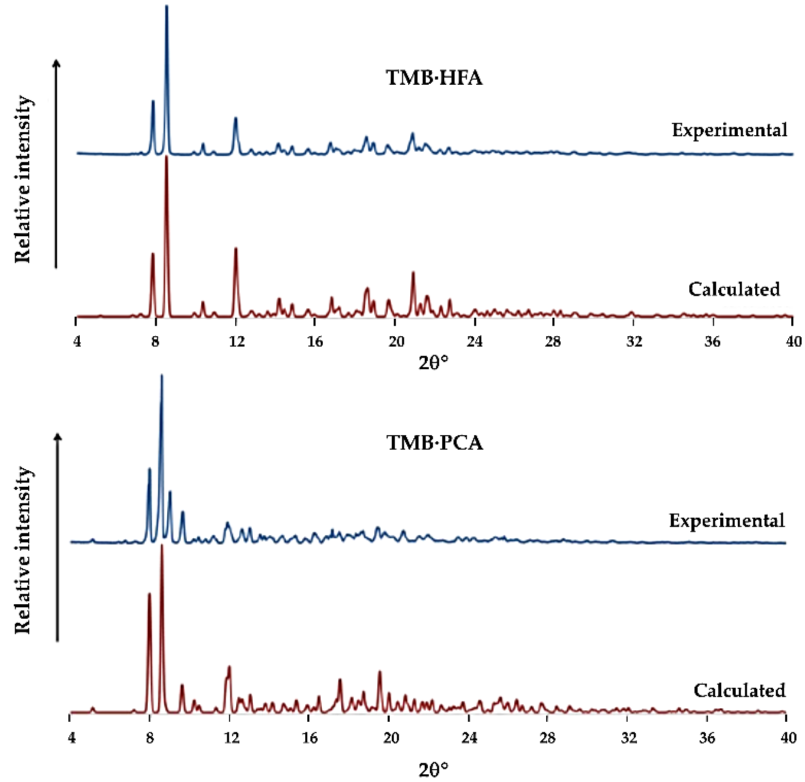

Figure S5. Experimental and calculated PXRD patterns for TMB·HFA and TMB·PCA.

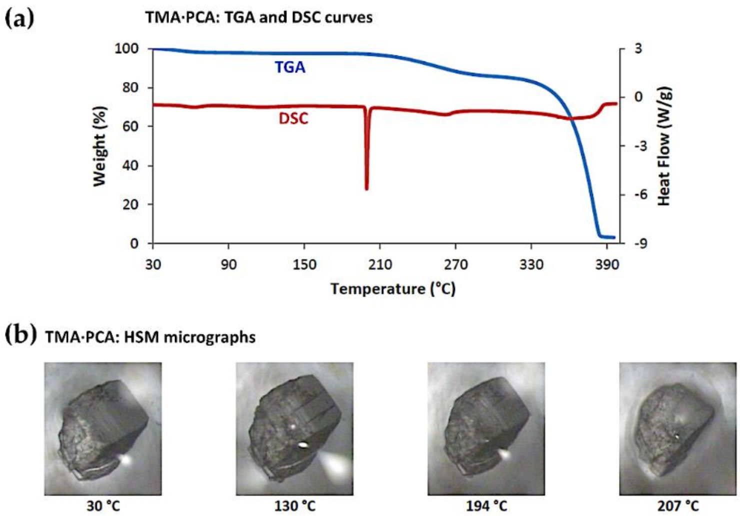

Figure S6. TGA and DSC traces (a) and HSM micrographs (b) for the complex TMA·PCA.

Table S4. Crystallographic data for TMA·PCA

	TMA·PCA
Complex Formula	$C_{54}H_{96}O_{30}·C_9H_8O_3·4.5H_2O$
Formula weight	1470.53
Crystal system	Orthorhombic
Space group	$P2_12_12_1$ (No. 19)
a / Å	15.952(2)
b / Å	18.792(2)
c / Å	25.491(3)
Volume / $Å^3$	7642(1)
Z	4
Calculated density / g cm^{-3}	1.278
μ (MoKα) / mm^{-1}	0.105
F (000)	3164
Temperature / K	173(2)
Crystal size / mm^3	0.28 x 0.32 x 0.45
Theta range scanned / °	1.9 < θ < 27.3
Index ranges	h: -11: 20; k: -19: 24; l: -27: 32
Total number of reflections	29033
No. of independent reflections	16977
No. of reflections with I > 2σ(I)	10586
No. of parameters	915
R_{int}	0.035
R_1(I > 2σ(I))	0.0751
wR_2(I > 2σ(I))	0.2139
S	1.022
Coefficients in weighting scheme	a = 0.1135, b = 3.4339
Δρ excursions / e $Å^{-3}$	-0.39, 0.66

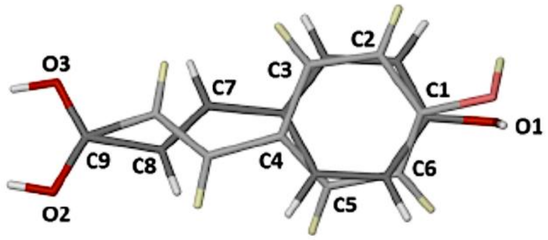

Figure S7. Disorder of the PCA molecule in the TMA·PCA complex.

Table S5. Geometrical parameters of the TMA molecule in the complex TMA·PCA.

Residue	r (Å)	D (Å)	a (°)	φ (°)	d (°)	D_3 (Å)	α (Å)	τ_1 (°)	τ_2 (°)
G1	4.097	4.230	123.6	117.1(4)	-11.6	3.353	0.133(3)	15.5(2)	5.7(1)
G2	4.345	4.206	117.7	117.9(4)	10.0	3.114	-0.192(3)	17.4(1)	14.4(1)
G3	4.341	4.253	117.4	118.3(5)	-3.1	3.296	0.101(3)	5.4(1)	5.3(1)
G4	4.060	4.387	125.3	116.3(4)	-3.3	3.478	0.044(3)	30.8(1)	9.9(1)
G5	4.385	4.078	116.6	117.6(5)	2.3	3.313	-0.104(3)	9.8(1)	7.0(1)
G6	4.338	4.439	118.3	117.7(4)	5.1	3.408	0.018(3)	18.6(2)	3.5(1)
Mean	4.26	4.27	119.8	117.5	6.4	3.33	0.105	16.3	7.6

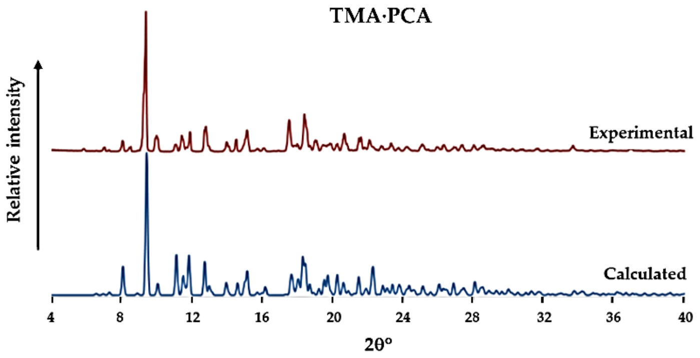

Figure S8. Experimental and calculated PXRD patterns for the complex TMA·PCA.

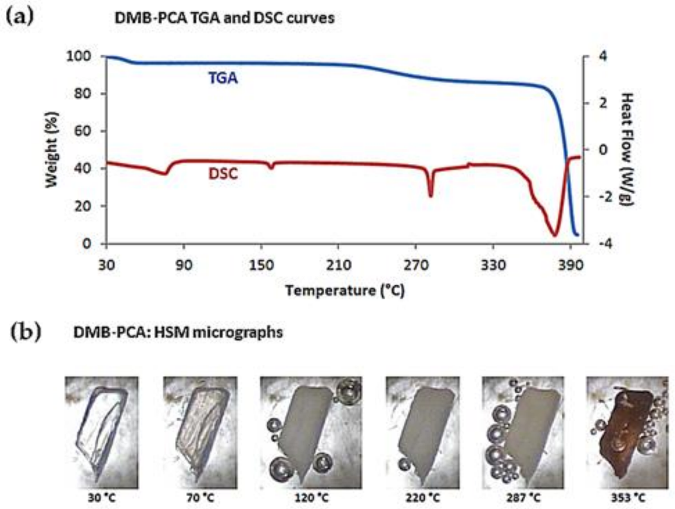

Figure S9. TGA and DSC traces (a) and HSM micrographs (b) for the complex DMB·PCA.

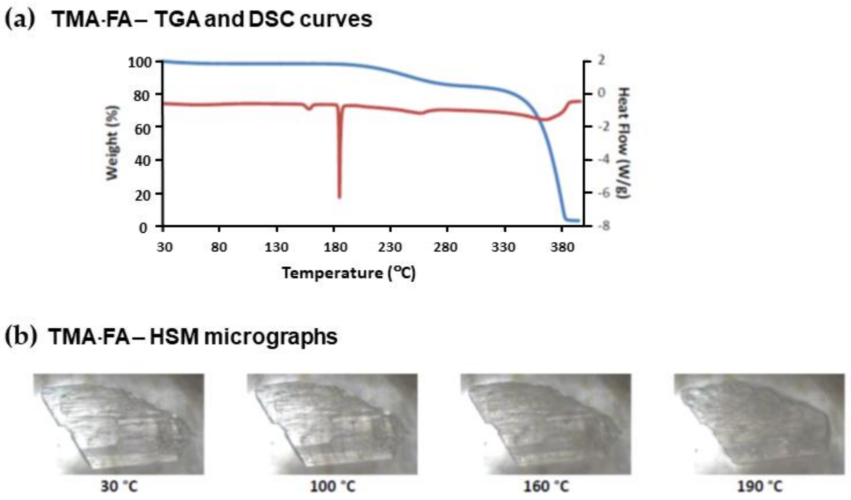

Figure S10. TGA and DSC traces (a) and HSM micrographs (b) for the complex TMA·FA.

Table S6. Crystallographic data for DMB·HFA and DMB·PCA

	DMB·HFA	DMB·PCA
Complex Formula	$C_{56}H_{98}O_{35}·C_{10}H_{12}O_4·3.85H_2O$	$C_{56}H_{98}O_{35}·C_9H_8O_3·3.2H_2O$
Formula weight	1596.89	1553.14
Crystal system	Orthorhombic	Orthorhombic
Space group	$P2_12_12_1$ (No. 19)	$P2_12_12_1$ (No. 19)
a / Å	14.732(1)	15.006(3)
b / Å	18.840(1)	19.033(4)
c / Å	29.205(2)	27.615(6)
Volume / $Å^3$	8106(1)	7887(3)
Z	4	4
Calculated density / g cm^{-3}	1.309	1.308
μ (MoKα) / mm^{-1}	0.110	0.109
F (000)	3426	3328
Temperature / K	173(2)	173(2)
Crystal size / mm^3	0.34 x 0.36 x 0.42	0.34 x 0.36 x 0.42
Theta ranges scanned / °	1.4 < θ < 28.4	2.8 < θ < 26.4
Index ranges	h: -19: 19; k: -25: 25; l: -39: 38	h: -18: 18; k: -23: 23; l: -34: 34
Total number of reflections	60872	103377
No. of independent reflections	20262	16089
No. of reflections with I > 2σ(I)	16933	13862
No. of parameters	1035	955
R_{int}	0.033	0.034
R_1(I > 2σ(I))	0.0499	0.0609
wR_2(I > 2σ(I))	0.1358	0.1698
S	1.010	1.038
Coefficients in weighting scheme	a = 0.0726, b = 3.0041	a = 0.0918, b = 5.9490
Δρ excursions / e $Å^{-3}$	-0.43, 0.59	-0.60, 0.82

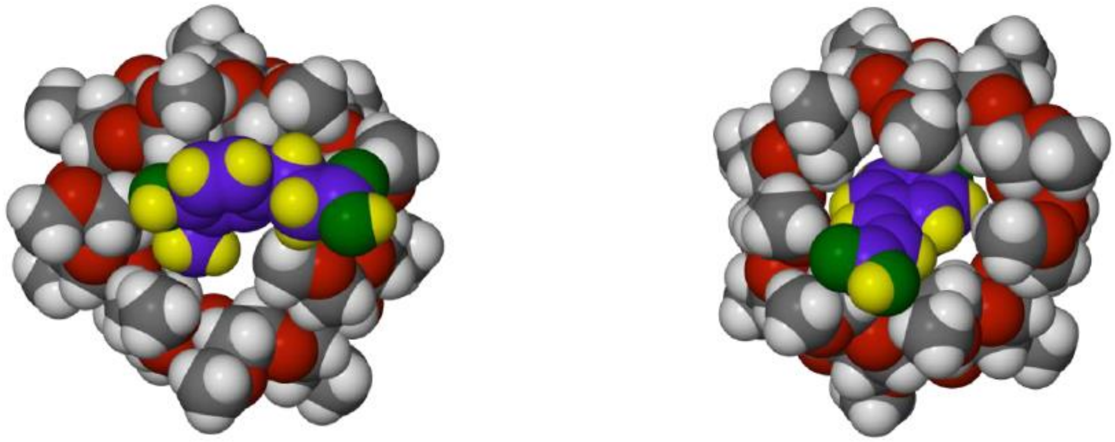

Figure S11. CPK space-filling representation of the inclusion complexes DMB·HFA (left) and DMB·PCA (right) viewed from the (narrow) primary sides of the host molecules.

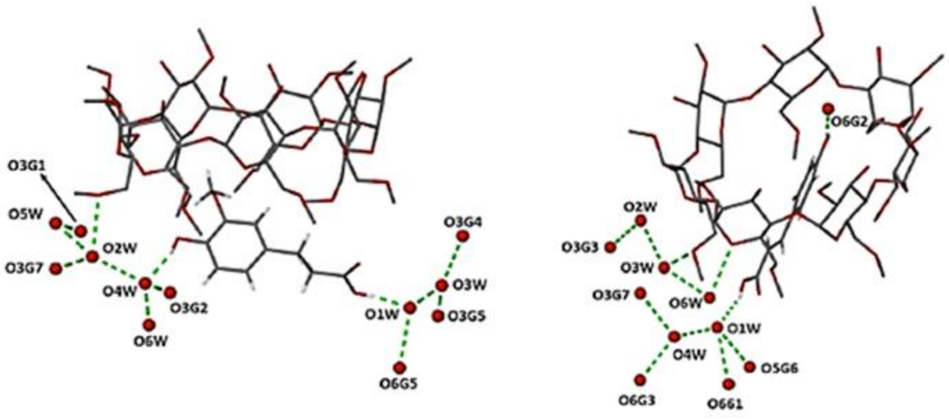

Figure S12. Principal H-bonds in DMB·HFA (left) and DMB·PCA (right). Red circles labelled OnW represent the oxygen atoms of water molecules. Atoms with labels of type OpGq (p = serial number, q in Gq = Glucose residue number q) are oxygen atoms of the host molecules. [Atom O6G2 (top right) is an acceptor of the hydrogen atom of the phenolic group of PCA. O6G2 belongs to a DMB molecule (not shown) that is partly included in the parent DMB molecule shown].

Table S7. Geometrical parameters of the DMB molecule in the complex DMB·HFA.

Residue	*r* (Å)	*D* (Å)	*a* (°)	*φ* (°)	*d* (°)	D_3 (Å)	*α* (Å)	$τ_1$ (°)	$τ_2$ (°)
G1	5.304	4.181	120.3	119.7(2)	-14.5	2.796(3)	-0.284(2)	17.3(1)	16.7(1)
G2	4.768	4.618	135.0	116.4(2)	2.3	2.868(4)	0.158(2)	13.3(1)	18.0(2)
G3	4.899	4.265	130.6	117.7(2)	8.3	2.865(3)	0.164(2)	4.0(1)	4.5(2)
G4	5.324	4.291	121.4	118.5(2)	-2.0	2.898(4)	-0.183(2)	12.4(1)	12.6(1)
G5	4.980	4.477	131.0	117.3(2)	-8.1	2.942(3)	-0.097(2)	19.8(1)	20.6(1)
G6	4.845	4.377	130.3	118.1(2)	3.4	2.787(3)	0.206(2)	9.9(1)	12.0(1)
G7	5.042	4.373	129.6	117.1(2)	10.1	2.843(3)	0.035(2)	3.8(1)	5.0(1)
**Mean**	**5.00**	**4.37**	**128.3**	**117.8**	**8.2**	**2.86**	**0.177**	**11.5**	**12.8**

Table S8. Geometrical parameters of the DMB molecule in the complex DMB ·PCA.

Residue	*r* (Å)	*D* (Å)	*a* (°)	*φ* (°)	*d* (°)	D_3 (Å)	*α* (Å)	τ_1 (°)	τ_2 (°)
G1	5.116	4.384	128.4	118.3(3)	3.2	2.776(5)	0.162(3)	12.9(1)	13.9(2)
G2	4.978	4.410	129.2	118.9(3)	7.1	2.826(6)	0.099(2)	8.6(1)	3.9(2)
G3	4.950	4.411	130.2	117.8(3)	-4.0	2.975(5)	-0.172(2)	20.1(1)	4.3(1)
G4	5.146	4.330	127.4	118.4(3)	-5.0	2.833(5)	-0.035(2)	18.4(1)	6.9(2)
G5	5.175	4.413	124.7	119.3(3)	4.4	2.816(5)	0.176(2)	5.8(1)	8.0(2)
G6	4.835	4.527	134.3	116.8(3)	6.5	2.894(5)	0.022(2)	12.0(1)	1.6(1)
G7	5.137	4.226	124.8	119.9(3)	-11.4	2.812(5)	-0.208(2)	19.6(1)	7.2(2)
**Mean**	**5.05**	**4.39**	**128.4**	**118.5**	**6.5**	**2.85**	**0.142**	**13.9**	**6.5**

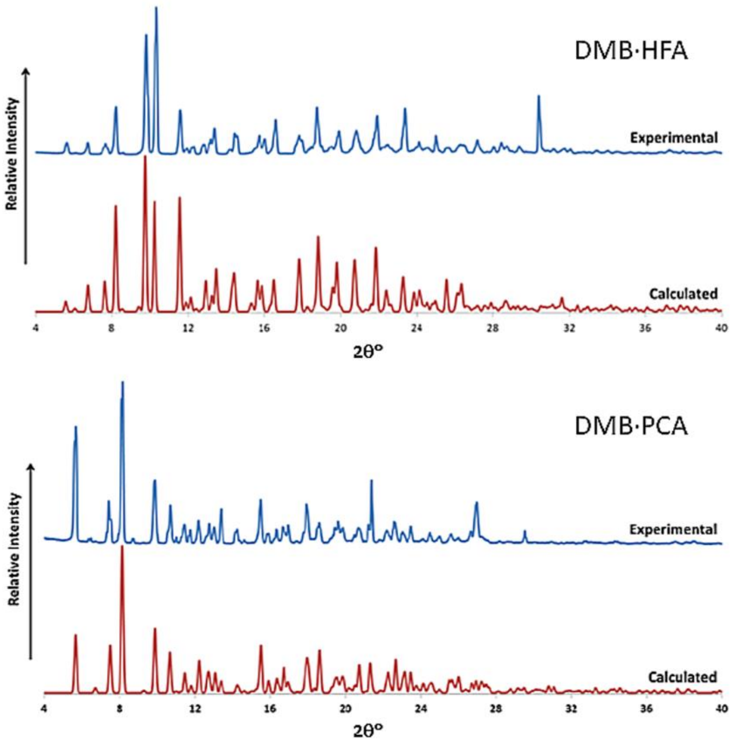

Figure S13. Experimental and calculated PXRD patterns for DMB·HFA and DMB·PCA.

Table S9. Crystallographic data for TMA·FA.

	TMA·FA
Complex Formula	$C_{54}H_{96}O_{30}\cdot C_{10}H_{10}O_4\cdot 1.25H_2O$
Formula weight	1442.00
Crystal system	Monoclinic
Space group	$P2_1$ (No. 4)
a / Å	11.123(2)
b / Å	23.701(5)
c / Å	14.310(3)
β / °	103.02(3)
Volume / $Å^3$	3675(2)
Z	2
Calculated density / g cm^{-3}	1.303
μ (MoKα) / mm^{-1}	0.106
F (000)	1549
Temperature / K	173(2)
Crystal size / mm^3	0.10 x 0.22 x 0.41
Theta range scanned / °	2.5 < θ < 26.4
Index ranges	h: -13: 13; k: -29: 29; l: -17: 17
Total number of reflections	58828
No. of independent reflections	14934
No. of reflections with I > 2σ(I)	13151
No. of parameters	923
R_{int}	0.036
R_1(I > 2σ(I))	0.0421
wR_2(I > 2σ(I))	0.1032
S	1.028
Coefficients in weighting scheme	a = 0.0488, b = 1.2570
Δρ excursions / e $Å^{-3}$	-0.40, 0.62

Table S10. Geometrical parameters of the TMA molecule in the complex TMA·FA.

Residue	*r* (Å)	*D* (Å)	*a* (°)	*φ* (°)	*d* (°)	D_3 (Å)*	*α* (Å)	τ_1 (°)	τ_2 (°)
G1	4.239	4.324	119.9	118.3(2)	-13.3	3.508	0.084(1)	40.1(1)	41.1(1)
G2	4.133	4.396	122.2	118.1(2)	17.7	3.536	-0.336(2)	3.5(1)	7.7(1)
G3	4.457	4.093	113.0	119.6(2)	-5.0	3.299	0.253(2)	9.2(8)	11.8(1)
G4	4.142	4.379	124.4	115.7(2)	-12.3	3.612	0.070(1)	35.8(1)	38.2(1)
G5	4.192	4.239	119.0	118.4(2)	17.2	3.456	-0.324(1)	6.8(1)	10.4(1)
G6	4.340	4.162	117.8	118.3(2)	-5.0	3.244	0.253(1)	4.9(1)	8.7(1)
**Mean**	**4.25**	**4.27**	**119.4**	**118.1**	**11.9**	**3.44**	**0.226**	**16.7**	**19.7**

*Average e.s.d. = 0.003 Å

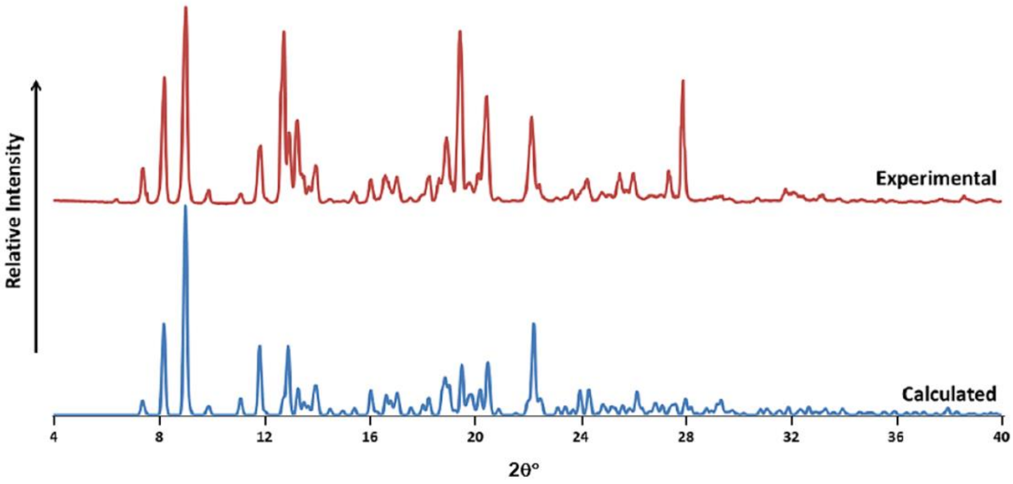

Figure S14. Experimental and calculated PXRD patterns for TMA·FA..

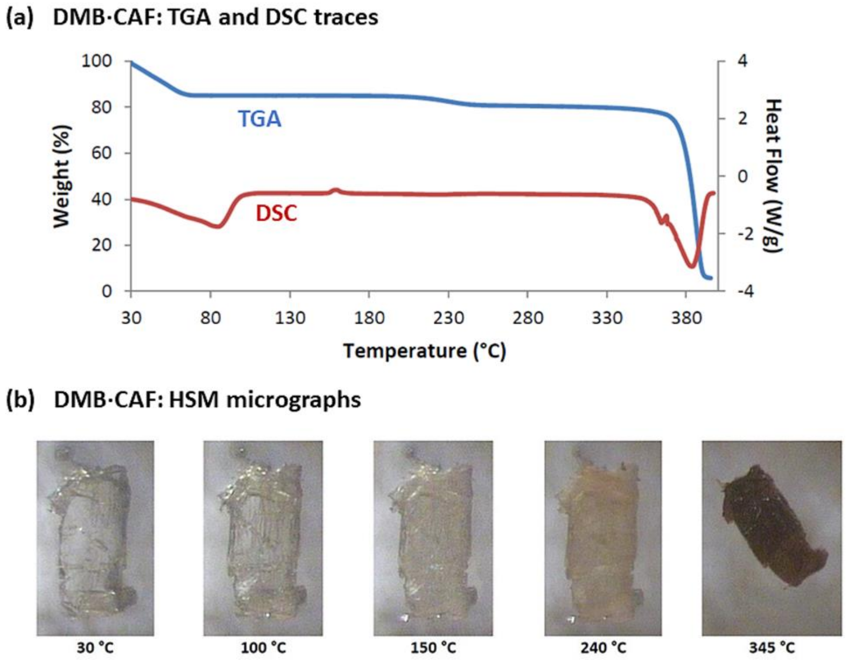

Figure S15. TGA and DSC traces (a) and HSM micrographs (b) for the complex DMB·CAF

Table S11. Crystallographic data for DMB·CAF and DMB·FA.

	DMB·CAF	DMB·FA
Complex Formula	$(C_{56}H_{98}O_{35})_2{\cdot}C_5H_8O_4{\cdot}12.6H_2O$	$(C_{56}H_{98}O_{35})_2{\cdot}C_{10}H_{10}O_4{\cdot}11.2H_2O$
Formula weight	3069.83	3058.63
Crystal system	Monoclinic	Monoclinic
Space group	$P2_1$ (No. 4)	$P2_1$ (No. 4)
a / Å	17.550(2)	17.567(4)
b / Å	25.851(2)	25.757(5)
c / Å	17.787(2)	17.847(4)
β / °	101.50(1)	100.97(2)
Volume / $Å^3$	7907(2)	7928(3)
Z	2	2
Calculated density / g cm^{-3}	1.289	1.272
μ (MoKα) / mm^{-1}	0.110	0.109
F (000)	3296	3284
Temperature / K	173(2)	173(2)
Crystal size / mm^3	0.20 x 0.22 x 0.51	0.18 x 0.25 x 0.45
Theta range scanned / °	$1.4 < \theta < 22.8$	$2.8 < \theta < 26.4$
Index ranges	h: -19: 19; k: -28: 28; l: -19: 19	h: -21: 21; k: -32: 32; l: -22: 22
Total number of reflections	51904	121634
No. of independent reflections	21330	32264
No. of reflections with $I > 2\sigma(I)$	18874	27296
No. of parameters	1818	1866
R_{int}	0.027	0.035
$R_1(I > 2\sigma(I))$	0.0559	0.0615
$wR_2(I > 2\sigma(I))$	0.1533	0.1784
S	1.028	1.021
Coefficients in weighting scheme	a = 0.0922, b = 5.2881	a = 0.1132, b = 3.3305
Δρ excursions / e $Å^{-3}$	-0.52, 0.73	-0.48, 1.08

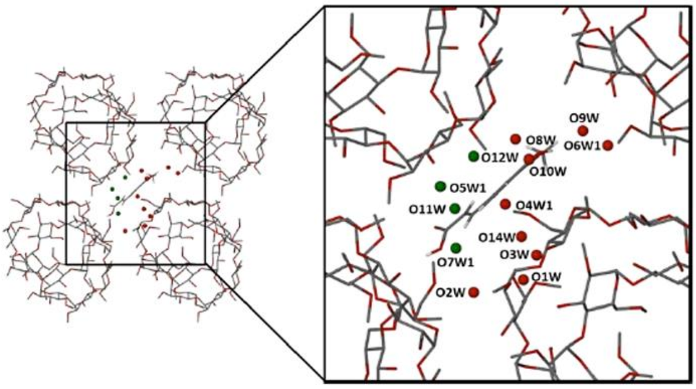

Figure S16. Packing in DMB·FA showing the interstitial channel created by four spiral columns in which the guest molecule FA and water molecules are located. The contents of the arbitrary square drawn on the left are magnified on the right. The view direction deviates slightly from being parallel with the crystal *b*-axis to reduce atomic overlap. Green spheres represent water oxygen atoms that are not directly H-bonded to the CDs.

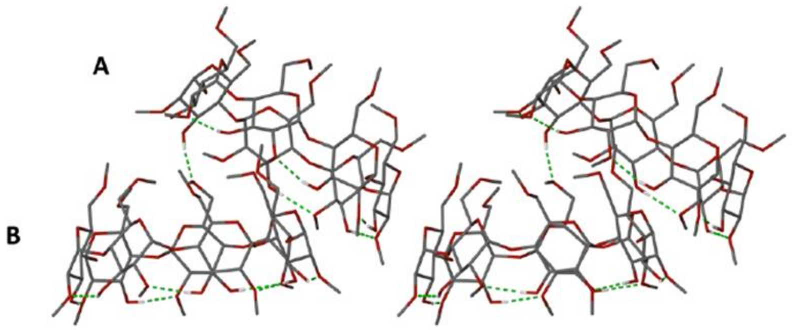

Figure S17. Stereodiagram showing the inclusion of two primary methoxyl groups of DMB molecule B into the cavity of molecule A *via* the secondary rim. For clarity, the only H atoms shown are those involved in H-bonding, namely those of type O2n ···H-O3(n-1), maintaining the round shape of the DMB molecules, and the strong H-bond O3A4-H···O6B6, linking the two CD molecules directly.

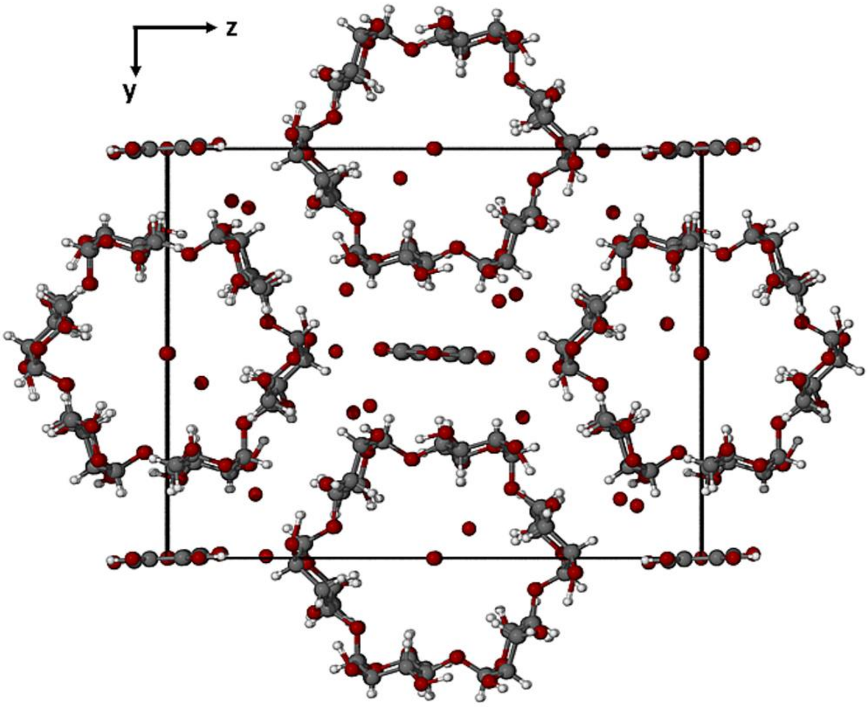

Figure S18. The [100] projection of the crystal structure of the complex α-CD·2,5-dihydroxybenzoic acid (refcode WIZQEB). The guest molecules are located in the interstitial space created by the surrounding columns of α-CD cmolecules. The isolated red spheres are oxygen atoms of water molecules.

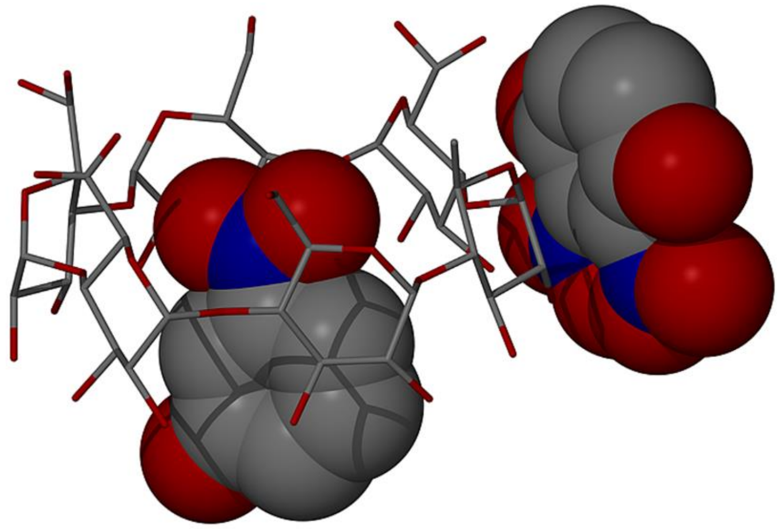

Figure S19. Simultaneous guest inclusion and non-inclusion in the α-CD·(*m*-nitrophenol)$_2$ complex (CSD refcode ACDMNP).
